# Immune gene diversity and *STING1* variants in shaping cancer immunity across different genetic ancestry populations

**DOI:** 10.1016/j.celrep.2025.116882

**Published:** 2026-01-21

**Authors:** Xiaowen Hu, Jie Huang, Jiao Yuan, Yanrong Sun, Yuxin Wang, Zhongyi Hu, Junjie Jiang, Zhiling Wang, Bingwei Wang, Meixiao Long, Kara N. Maxwell, Yi Fan, Janos L. Tanyi, Kathleen T. Montone, Hongzhe Li, Sarah H. Kim, Katherine L. Nathanson, Timothy R. Rebbeck, Susan M. Domchek, Robert H. Vonderheide, Lin Zhang

**Affiliations:** 1 Center for Women’s Health and Reproductive Medicine, University of Pennsylvania, Philadelphia, PA 19104, USA; 2 Department of Obstetrics and Gynecology, University of Pennsylvania, Philadelphia, PA 19104, USA; 3 Division of Hematology, Department of Internal Medicine, Ohio State University, Columbus, OH 43210, USA; 4 Division of Hematology and Oncology, Department of Medicine, University of Pennsylvania, Philadelphia, PA 19104, USA; 5 Abramson Cancer Center, University of Pennsylvania, Philadelphia, PA 19104, USA; 6 Department of Radiation Oncology, University of Pennsylvania, Philadelphia, PA 19104, USA; 7 Department of Pathology and Laboratory Medicine, University of Pennsylvania, Philadelphia, PA 19104, USA; 8 Department of Biostatistics, Epidemiology and Informatics, University of Pennsylvania, Philadelphia, PA 19104, USA; 9 Division of Translational Medicine and Human Genetics, Department of Medicine, University of Pennsylvania, Philadelphia, PA 19104, USA; 10 Basser Center for BRCA, University of Pennsylvania, Philadelphia, PA 19104, USA; 11 Dana-Farber Cancer Institute and Harvard T.H. Chan School of Public Health, Boston, MA 02115, USA; 12 These authors contributed equally; 13 Lead contact

## Abstract

As human populations migrated to diverse geographical regions, they encountered varying pathogens, leading to pronounced natural selection pressures on the immune system. Analysis of non-synonymous single-nucleotide polymorphisms (nsSNPs) across major geographically structured populations showed greater variation in immune-related genes than in non-immune genes, consistent with pathogen-driven selection, whereas cancer-related genes exhibited lower variation, reflecting the evolutionary conservation of critical cellular functions. We prioritized nsSNPs in pattern recognition receptor genes based on population diversity and their association with type I interferon (IFN) activity. Among the top-ranked variants were rs11554776, rs78233829, and rs7380824 in *STING1*, which demonstrated functional impacts on intrinsic cGAS-STING1-IFN signaling in cancer cells and potential influences on tumor immunity. We further conducted a genome-wide characterization of nsSNPs in immune-related genes across genetic ancestry populations and established a publicly accessible database. Our study suggests that genetic ancestry-related germline variations may influence cancer immunity and treatment, supporting their consideration in personalized medicine.

## In brief

Hu et al. analyzed non-synonymous SNPs across diverse human populations and revealed divergent evolutionary pressures on immune- and cancer-related genes. By integrating population diversity with functional evaluation, they identified *STING1* variants as modulators of interferon signaling. Their findings suggest that germline variations shaped by genetic ancestry may influence cancer immunity.

## Graphical Abstract

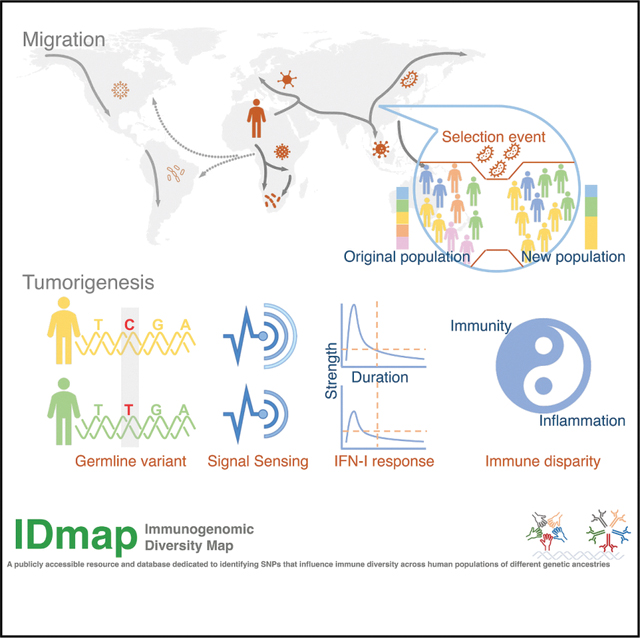

## INTRODUCTION

Modern humans originated in Africa approximately 200,000 years ago and, over the past 50,000 to 100,000 years, migrated out of Africa and dispersed across the globe.^1^ As humans ventured into diverse geographical regions, they encountered varied pathogens, food sources, and climate conditions, necessitating adaptation.^1^ The immune system, particularly the innate immune system, faced critical selection pressures, likely surpassing those experienced by other biological systems, as many infectious diseases posed lethal threats to early humans.^1–5^ These selective pressures ultimately contributed to the population- or region-specific germline genetic variants observed in the human immune system today, which influence the variable intensity and timing of responses to specific pathogens.^1–5^ Subsequent migrations, such as those following the discovery of the New World, further increased the diversity of immune system genes. Differences in immune system composition among distinct populations, shaped during human migration, not only underpin varying responses to pathogen infections but also influence other immune-related conditions, such as autoimmune and inflammatory diseases.^1–5^ Importantly, population-associated immune gene variants continue to influence clinical outcomes today.

Cancer health disparities have remained a persistent challenge in the United States.^6–8^ It has been well established that, in addition to socioeconomic, cultural, and environmental factors, biological differences related to genetic ancestry also significantly contribute to these disparities.^9–16^ For example, through the integration of large-scale cancer genomic profiles from The Cancer Genome Atlas (TCGA), we identified distinct genomic instability profiles and genomic alteration spectra among tumors originating in diverse genetic ancestries.^9^ Ancestry-related diversity in immune response to tumorigenesis has been extensively documented, exerting a significant influence on tumor progression and treatment outcomes^17,18^ attributed to distinct patterns of antitumor immunity and differing prevalence of inflammatory microenvironments. Both our research^19^ and that of others^20–22^ has reported that prostate cancers from patients of pre-dominantly African ancestry frequently demonstrate heightened levels of inflammatory signaling compared to patients of European ancestry. Further, clinical observation suggests that African Americans (AAs) may display unique immune therapy responses^23,24^ and differential predictive biomarkers for immune therapy.^24–26^

Non-synonymous coding single-nucleotide polymorphisms (nsSNPs) are germline genetic variants that alter amino acid sequences in proteins.^27^ Distinct from synonymous, non-coding, and intronic SNPs, nsSNPs hold the potential to directly affect protein structure, stability, post-translational modifications, cellular localization, and interactions, consequently influencing protein function. Many rare non-synonymous variants, especially those discovered in genes related to genome integrity and DNA damage repair pathways, have been shown to be associated with an increased risk of cancer.^28–31^ Due to the strong selective pressures of human evolution, common nsSNPs generally are considered to be neutral or to have only minor phenotypic effects.^32^ Beyond their involvement in cancer susceptibility, the impact of nsSNPs on tumor progression, particularly in the context of tumor immunity and inflammation, remains poorly understood. Notably, nsSNPs have demonstrated their critical involvement in pathogen infection,^30,33^ autoimmune diseases,^30,34^ and immune-mediated mechanisms in non-malignant conditions.^30,35^ Numerous functional nsSNPs have been associated with variation in immune response strength and duration, particularly within genes encoding innate immune response pathway proteins.^36,37^ Large-scale analyses of cancer genomes have suggested that common germline genetic variants can influence the functional characteristics of the tumor immune microenvironment.^38–43^

## RESULTS

### Immune-related genes exhibit increased F_ST_ values for nsSNPs, whereas cancer-related genes display lower F_ST_ values

**Figure 1.**
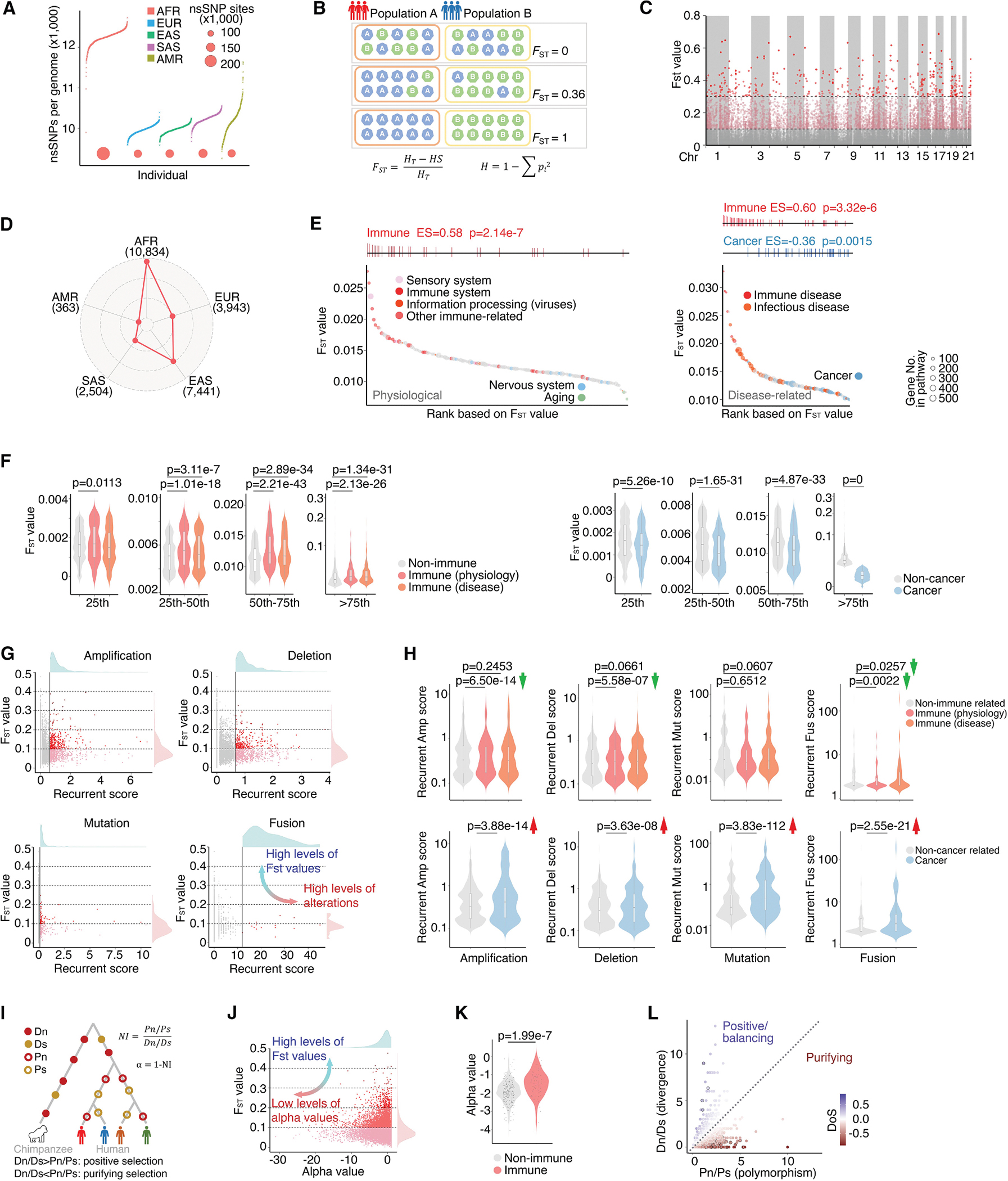
Immune-related genes exhibit increased F_ST_ values for nsSNPs, whereas cancer-related genes display lower F_ST_ values (A) Each major geographically structured population is depicted by the number of nsSNPs per genome. Each dot corresponds to an individual and reflects the number of nsSNPs. Dots are colored by their corresponding ancestry. The size of the lower bubbles indicates the total number of nsSNP sites within the respective population. (B) Diagram illustrating the approach that estimates F_ST_ value. (C) The distribution of global F_ST_ values for nsSNPs across the human genome is represented by colored dots. Dot colors indicate different F_ST_ value ranges: red, >0.3; pink, between 0.1 and 0.3; gray, <0.1. (D) Radar plot illustrates the number of genes with F_ST_ values exceeding 0.1 in a given population compared to the remaining populations. (E) The bubble plots show mean F_ST_ values for each pathway depicted across physiological (left) and disease-related (right) pathways. Pathways within each group are ranked by the mean F_ST_ value. Bubble size represents the number of genes in each pathway, and color denotes selected pathways. Top displays gene set enrichment analysis results for pathways significantly enriched for higher or lower F_ST_ values. (F) Violin plots illustrate F_ST_ values of genes in immune-related (left) and cancer-related pathways (right) compared to unrelated pathways, respectively. Comparisons are grouped by F_ST_ values: <25th percentile, 25th to 50th percentile, 50th to 75th percentile, and >75th percentile. (G) Associations between F_ST_ values and recurrent scores of amplification, deletion, mutation, and fusion. Red dots denote genes surpassing respective genomic score thresholds. Density plots represent the distributions of F_ST_ values (red) and recurrent scores (green). (H) Violin plots illustrate genomic recurrent scores of genes in immune-related (top) and cancer-related pathways (bottom), compared to unrelated pathways. Green and red arrows indicate significantly lower and higher genomic recurrent scores in the selected gene groups, respectively. (I) The schematic illustrates the McDonald-Kreitman test. (J) Correlation between F_ST_ and alpha values for autosomal protein-coding genes. Density plots show the distributions of F_ST_ (right) and alpha values (top) for the genes under consideration. The red arrow indicates the direction of low alpha values and the green arrow indicates the direction of high F_ST_ value. (K) Violin plot represents alpha values between immune-related and non-immune-related pathways. (L) Distribution of Dn/Ds and Pn/Ps ratios for immune-related pathways. Color denotes the type of selection, while intensity reflects the direction of selection (DoS), calculated as DoS = (Dn/(Dn + Ds)) – (Pn/(Pn + Ps)). A positive DoS value indicates positive selection; a negative value indicates purifying selection. Genes with significant scores are accentuated with black circles.

nsSNP information was collected from the 1000 Genomes Project,^44^ covering 2,548 individuals representing 26 populations across Africa (AFR), East Asia (EAS), Europe (EUR), South Asia (SAS), and the Americas (AMR; Table S1). A total of 580,277 nsSNPs were identified, accounting for 0.79% of all SNPs and 22.40% of exon-located SNPs (Figure S1A). Consistent with previous reports on overall SNPs,^44^ substantial divergence in nsSNP frequencies among populations was observed (Figures 1A and S1B; Tables S2 and S3). To assess the variations in nsSNPs among major geographically structured populations, we utilized Wright’s F statistics^45,46^ to estimate autosomal genome-wide fixation index (F_ST_) at both the SNP and the gene levels (Figure 1B). We found that 7,662 nsSNPs exhibited high F_ST_ values (>0.1), with 439 nsSNPs demonstrating F_ST_ values surpassing 0.3, reflecting significant variation among populations (Table S4). These population-diverse nsSNPs are randomly distributed across the human genome (Figure 1C), and a similar pattern was also observed at the gene level for nsSNPs (Figure S1C). When conducting Wright’s F-statistics analysis of individual populations, we found that the five super populations showed distinct diversity, with AFR exhibiting the highest divergence (Figures 1D and S1D).

The Kyoto Encyclopedia of Genes and Genomes (KEGG)^47^ was used to classify the biological pathways of genes, resulting in a total of 341 pathways, comprising 249 physiological pathways (including 84 associated with metabolism) and 92 pathological pathways (Figure S1E; Table S5). Following the assignment of genes to their respective pathways, we conducted an analysis of the average F_ST_ values for nsSNPs within these genes across each pathway. Immune-related pathways displayed significantly higher F_ST_ values compared to other physiological pathways (*p* = 2.14e–7, enrichment score [ES] = 0.58; Figure 1E). Conversely, pathways associated with aging or the nervous system exhibited lower F_ST_ values. Most essential metabolic pathways, such as those involved in fat and protein metabolism, demonstrated low F_ST_ values indicative of strong evolutionary conservation; however, certain metabolic pathways, including iron and acid metabolism, exhibited high F_ST_ values (Figure S1F). Last, across disease-related pathways, immune-related disease pathways consistently showed significant enrichment, with high F_ST_ values (*p* = 3.32e–6, ES = 0.60; Figure 1E). Despite distinct frequencies of certain somatic alterations being observed among different genetic ancestry populations,^9–16^ cancer-related pathways display significantly lower F_ST_ values (*p* = 0.0015, ES = −0.36; Figure 1E), possibly due to the essential functional role of genes within these pathways in maintaining cellular fitness. In summary, across all KEGG-defined pathways in humans, immune-related pathways (both physiology and disease) exhibited significantly higher F_ST_ values (*p* = 2.62e–5 and *p* = 2.39e–6, respectively) compared to other pathways, whereas cancer-related pathways displayed lower F_ST_ values (*p* = 0.0002). Notably, upon categorizing genes into different groups based on F_ST_ values, we found that those in the higher percentiles (e.g., >50th) exhibited more significant differences (Figure 1F). With the significantly low F_ST_ values observed among the genes within KEGG cancer pathways, we investigated the relationship between F_ST_ values and recurrent genomic alterations in cancer. The recurrent genomic alteration scores^48,49^ for each gene were estimated across the TCGA cohort (Table S6), providing a quantification of whether specific genes’ somatic copy-number alterations (SCNAs), mutations, and fusions occur at a statistically higher frequency than the background within cancer genomes (Figures S1G–S1I). We observed that genes with higher recurrent alteration scores for SCNAs, mutations, and fusions had lower F_ST_ values (Figure 1G), further demonstrating that cancer-related genes, characterized by higher recurrent alteration scores, tend to have reduced diversity in nsSNPs across different populations. Notably, immune-related genes displayed moderately lower recurrent genomic alteration scores in comparison with non-immune-related genes (Figure 1H), indicating their potential role in adaptive functions during tumorigenesis rather than being the primary driver events. As expected, cancer-related genes exhibited strong and significantly higher recurrent genomic alteration scores in comparison with non-cancer genes.

The differential F_ST_ values observed for nsSNPs across various biological pathways suggest that the genes within these pathways have likely undergone different pressures and forms of natural selection. To quantitatively analyze the nature of selection acting on these genes, we employed the McDonald-Kreitman test^50^ to compare the ratio of synonymous and non-synonymous substitutions between the genomes of humans and chimpanzees (Figure 1I). We observed that the genes with lower F_ST_ values always show stronger purifying selection, which stabilizes genes through the removal of deleterious genetic polymorphisms that arise through random mutations (Figure 1J). As expected, immune-related pathways exhibited higher alpha values compared to non-immune-related pathways (Figure 1K), suggesting that immune pathways were subjected to less purifying selection pressure during evolution. Nevertheless, among the genes encoding proteins in immune-related pathways by KEGG, the alpha value and neutrality index (NI) also showed remarkable variation (Figure 1L), suggesting differential selection pressures. Within immune pathways, the genes encoding chemokines and cytokines showed higher alpha values, whereas the genes encoding adhesion molecules, kinases, and enzymes exhibited lower alpha values (Figure S1J).

### nsSNPs in PRR pathway genes are associated with diverse intrinsic interferon activity in cancer cells

**Figure 2.**
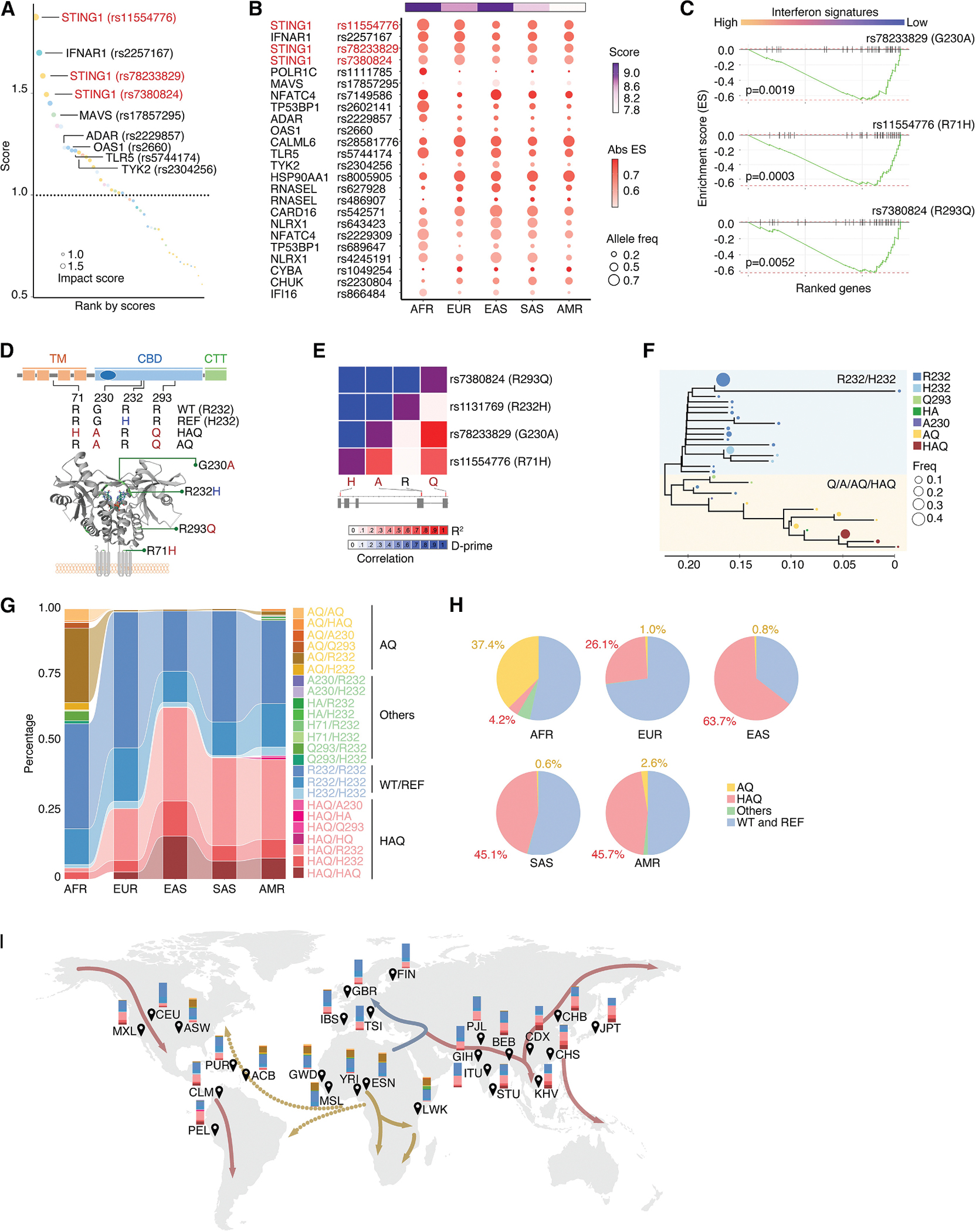
nsSNPs in PRR pathway genes are associated with diverse intrinsic interferon activity in cancer cells (A) Impact scores for the top-ranked nsSNPs (impact score >1) and their corresponding genes. Each nsSNP is ranked based on its impact score. The size of the bubble denotes the level of the impact score, and the color signifies its functional category. (B) Bubble plot presents the top 24 nsSNPs across five super populations. The size of each bubble indicates the frequency of alleles enriched for interferon signature, and the intensity of red denotes the absolute value of the enrichment score obtained from gene set enrichment analysis (GSEA). The top bar indicates the overall strength of enrichment and allele frequency in each super population. (C) Enrichment plots show the interferon signature in cancer cell lines carrying three nsSNPs in the *STING1* gene. (D) Illustration of the human STING1 protein structure. (E) Matrix of pairwise linkage disequilibrium between four nsSNPs in the *STING1* gene. Colored bars represent R^2^ (red) and D′ (blue) statistics. (F) Phylogenetic tree illustrates the evolutionary history of *STING1* haplotypes in humans. Haplotypes in *STING1* were estimated using 1000 Genomes Project samples. The color of the circle represents the major genotype in this haplotype; the size of the circle indicates the frequency of each haplotype in human populations according to the 1000 Genomes Project. (G) Alluvial plot illustrates the distribution of distinct *STING1* haplotypes among five super populations in the 1000 Genomes Project cohort. Colors represent the different haplotypes for the *STING1* gene. (H) Pie plots illustrate the distribution of four major *STING1* haplotype groups among five super populations in the 1000 Genomes Project cohort. Colors represent the different haplotype groups for the *STING1* gene. (I) A worldwide map displays the distribution of *STING1* haplotypes across 26 populations. Each bar within the map represents the distribution of *STING1* haplotypes within a specific population group, with arrows indicating the paths of human evolution.

Characterizing the functional impact of nsSNPs on tumor immunity is challenging due to the complexity of immune responses. Intrinsic type I interferon (IFN-I) activity in tumor cells, typically triggered by endogenous cytoplasmic DNA or double-stranded RNA, can be measured by IFN-I-stimulated gene (ISG) expression.^51^ Large-scale collections of cancer cell lines with detailed genetic profiles, such as DepMap,^52^ provide a unique and simplified model for proof-of-concept studies; however, they have inherent limitations, such as insufficient sample size for conducting classical genome-wide association studies (GWASs). To address this challenge, we employed a systems biology approach to functionally rank candidate nsSNPs, enabling their prioritization for downstream validation. Instead of relying on a classical GWAS approach, which establishes statistical thresholds for genotype-phenotype correlations, we implemented a ranking strategy to prioritize SNPs potentially associated with key biological functions, such as intrinsic IFN-I activity. This integrative prioritization method allows us to identify nsSNPs that are more likely to have functional effects, even when statistical power is limited. Our analysis focused on five KEGG pathways within the pattern recognition receptor (PRR) network, which includes germline-encoded sensors that detect pathogen-associated molecular patterns and initiate IFN-I signaling.^53–58^ We identified 324 autosomal genes encoding proteins in the PRR pathways (PRR-Genes, Table S7), categorized into eight functional groups (Figure S2A), with no significant differences in nsSNP minor allele frequency (MAF) or F_ST_ values across these groups (Figures S2B and S2C). RNA sequencing (RNA-seq) profiling demonstrated that a large proportion of PRR-Genes (85.09%) were intrinsically expressed in non-hematological cancer cell lines (Figure S2D), mirroring their wide-spread expression in normal cells as part of innate immune defenses. Through a four-step strategy, we ranked nsSNPs in PRR-Genes for their potential influence on intrinsic IFN-I signaling (Figure S2E). Twenty-four nsSNPs from 18 PRR-Genes showed a high impact score (>1), including those in *STING1*, *IFNAR1*, *MAVS*, *ADAR*, *OAS1*, *TLR5*, and *TYK2* (Figures 2A, 2B, and S2F; Table S9). rs11554776 (R71H) in *STING1* had the highest impact score, and another two nsSNPs in *STING1*, rs78233829 (G230A) and rs7380824 (R293Q), also displayed top impact scores. These nsSNPs demonstrated a significant correlation with decreased intrinsic ISG signature in cancer cells (Figure 2C), consistent with previous reports on these variants in immune cells.^59–61^ Supporting their potential roles in STING1 function, the G230A and R293Q variants are integral to the cyclic di-GMP-binding domain (CBD), with G230A at the peak and R293Q at the foundation; the R71H variant is embedded within the transmembrane domain, situated in a contiguous cytoplasmic loop proximal to the lower region of the binding pocket (Figure 2D). Notably, G230A and R293Q variants are in linkage disequilibrium and commonly co-occur with R71H, constituting haplotypes known as AQ or HAQ (Figure 2E). Phylogenetic analyses suggested that A230 is the ancestral allele from which the AQ and subsequently the HAQ haplotypes evolved (Figure 2F). This pattern suggests that a single-nucleotide substitution may have given rise to the A230, followed by additional mutations leading to AQ and HAQ haplotypes. The distribution of these haplotypes across diverse ancestral backgrounds indicates that it’s likely they occurred during human population divergence. The emergence of AQ and HAQ may have been shaped by selective pressures, potentially related to pathogen exposure or immune modulation, given STING1’s role in innate immunity. Whereas 31 haplotypes were identified in human populations (Table S10), the predominant haplotypes of *STING1* include the R232 (wild type; WT), R232H (reference; REF), HAQ (R71H, G230A, and R293Q), and AQ (G230A and R293Q) alleles (Figures 2F and 2G). R232, the haplotype commonly used in functional studies of *STING1*, is referred to as the WT haplotype, while R232H, the haplotype present in the reference genome, is referred to as the REF haplotype. Notably, significant population stratification is evident for the HAQ and AQ alleles. The AQ haplotype is most prevalent in AFR populations, consistent with the origin of modern humans in Africa and the retention of ancestral variants. In contrast, the HAQ haplotype is rare in AFR populations but predominantly occurs in EAS, SAS, and AMR (Figures 2G–2I), suggesting regional expansion following human migration and possible positive selection in the EAS population. Conversely, the WT and REF variant distributions reflect global human population patterns, with increased frequency in EUR and reduced frequency in the EAS population, which may indicate population-specific selective pressures or genetic drift. These observed haplotype distributions align with known out-of-Africa migration routes and suggest that environmental or pathogenic exposures may have shaped STING1 genetic variation through local adaptation. These observations were further confirmed through analysis conducted at the Genome Aggregation Database (gnomAD),^62^ which encompasses genetic information from more than 140,000 individuals (Figure S2G).

### *STING1* variants functionally influenced intrinsic cGAS-STING1-IFN signaling in cancer cells

**Figure 3.**
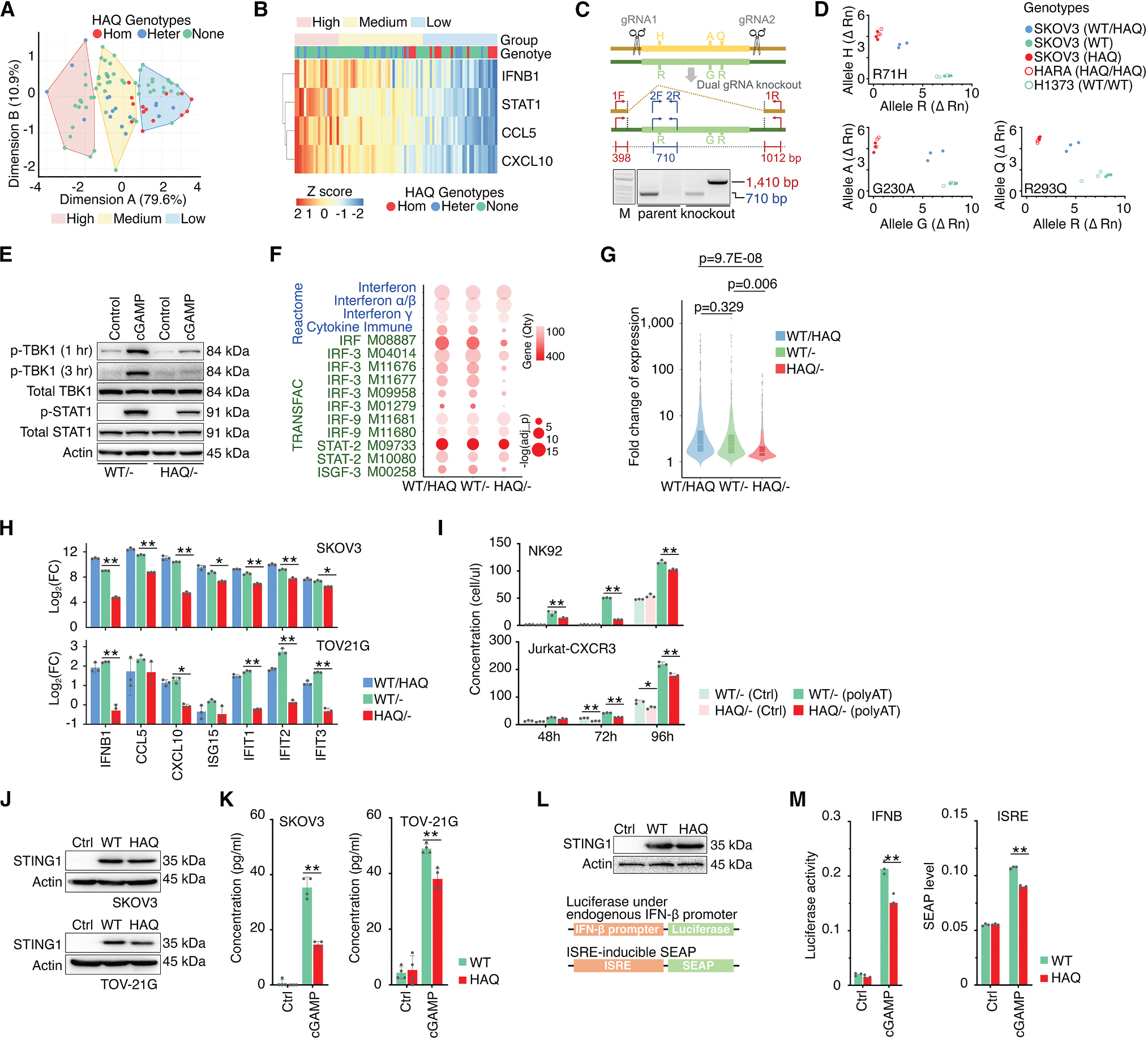
*STING1* variants functionally influence intrinsic cGAS-STING1-IFN signaling in cancer cells (A) The K-means clustering algorithm was used to group the cancer cell lines based on their similarity of IFN-β and ISG expression in response to cGAMP treatment (10 μg/mL, 30 min). The *STING1* haplotypes of each cell line are indicated using color coding. (B) Heatmap plot illustrates the changes in expression levels of IFN-β and ISGs following cGAMP treatment. The K-means group and *STING1* haplotypes of each cancer cell line are represented by color codes at the top of the heatmap. (C) Illustration of the dual gRNA/CRISPR strategy used to knock out an entire *STING1* gene allele in the *STING1* heterozygous cancer cell lines. (D) rhPCR-based allelic discrimination was used for genotyping the *STING1* gene in the collected clones. The normalized reporter signals (Rn) for allele 1 (FAM) and allele 2 (VIC) were plotted on the *x* and *y* axes, respectively. The allelic discrimination plot shows three distinct genotype clusters, including individuals homozygous (depicted in red) and heterozygous (shown in green) for the reference allele and those homozygous for the alternate allele (represented in blue). The homogeneous HAQ/HAQ line HARA and WT/WT line H1373 served as genotyping controls. (E) Western blot analysis of p-TBK1 and p-STAT1 was conducted in isogenic SKOV3 cells carrying either WT or HAQ *STING1*, following treatment with 10 μg/mL cGAMP. (F) Bubble plot illustrates the results of gene set enrichment analysis (GSEA) for the upregulated genes identified by RNA-seq following treatment with 10 μg/mL cGAMP for 30 min in isogenic SKOV3 cells carrying WT/HAQ, WT, or HAQ *STING1*. The IFN gene sets were obtained from Reactome, and the transcription-factor-regulated gene sets were sourced from TRANSFAC. The bubble size corresponds to the −log (adjusted *p* value), and the color intensity indicates the number of genes within each gene set. (G) Violin plot shows the fold changes of the commonly upregulated genes identified by RNA-seq after treatment with 10 μg/mL cGAMP for 30 min in isogenic SKOV3 cells carrying WT/HAQ, WT, or HAQ *STING1*. (H) The fold changes in expression levels of IFN-β and ISGs, as measured by RT-qPCR, following treatment with 10 μg/mL cGAMP for 30 min in isogenic SKOV3 and TOV21G cells carrying WT/HAQ, WT, or HAQ *STING1*. (I) The numbers of T cells (Jurkat-CXCR3, top) and NK cells (NK92, bottom) that migrated to the lower compartment, where isogenic SKOV3 cells were pretreated with 5 μg/mL poly(dA:dT) for 4 h. (J) WT or HAQ *STING1* cDNA was transduced into SKOV3 or TOV21G cells in which the endogenous *STING1* had been knocked out using CRISPR. The expression of STING1 protein was subsequently detected through western blots. (K) The levels of IFN-β expression measured by ELISA following treatment with 10 μg/mL cGAMP for 30 min in SKOV3 and TOV21G cells transduced with either WT or HAQ *STING1*. (L) WT or HAQ *STING1* cDNA was transduced into HEK293 reporter cells, which lacked endogenous STING1 expression and contained dual reporters. The expression of STING1 protein was subsequently detected through western blots. (M) The activities of ISRE and hIFN-β reporters after treatment with 10 μg/mL cGAMP for 30 min in 293-Dual Null reporter transduced with either WT or HAQ *STING1*. Data are represented as means ± SD. *p < 0.05 and **p < 0.01.

To confirm our genotype calling in cancer cell lines obtained through high-throughput profiling, the RNase H-dependent PCR (rhPCR; Figure S3A)^63^ was chosen for SNP validation.^64,65^ We conducted SNP genotyping on a randomly selected panel of cancer cell lines (*n* = 34), verifying prior genotype calls (Figure S3B). To investigate the functional impact of *STING1* variants on cancer cell intrinsic IFN-I signaling, we exposed these cells to cGAMP, a second messenger that directly activates STING1, and assessed the consequent induction of IFN-I in cells by quantifying IFN-β and ISG expression using RT-qPCR. Subsequent K-means clustering, based on the expression of cGAMP-stimulated ISGs, divided the cells into three groups (Figure 3A). Cell lines with non-HAQ *STING1* haplotypes were found to be evenly distributed across all clusters. In contrast, the majority of HAQ-homozygous cell lines were concentrated in the low-response cluster, and HAQ-heterozygous cell lines were predominantly observed in the low- and medium-response clusters (Figures 3A and 3B). This distribution indicated that the HAQ haplotype is associated with a reduced response to cGAMP-induced IFN-I stimulation. To further confirm this finding, we generated isogenic cancer cell lines with different *STING1* haplotypes. Because the HAQ haplotype consists of three closely linked nsSNPs within a sizable chromosomal region spanning 4,096 bp, conventional CRISPR-based base editing methods were ineffective for simultaneous editing of all three SNPs. Thus, we selected *STING1* heterozygous cancer cell lines with the WT/HAQ haplotypes and employed two guide RNAs (gRNAs) to disrupt the *STING1* gene allele, thereby generating isogenic cancer cell lines that exclusively carried either the WT or the HAQ allele (Figures 3C and 3D). We found that, after cGAMP treatment, HAQ cells exhibited remarkably lower activation of STING1 downstream signaling, including TBK1 and STAT1 phosphorylation, compared to WT cells (Figures 3E and S4A). RNA-seq analysis further demonstrated that, although cGAMP stimulation can significantly upregulate genes in the IFN pathways and those containing IRF and STAT/ISGF-3 promoter-binding motifs in both WT and HAQ cells, the magnitude of the effects and the fold upregulation were significantly weaker in HAQ cells compared to WT (Figures 3F and 3G; Table S11). This observation was further confirmed by RT-qPCR in two independent cancer cell lines (Figure 3H). Importantly, Transwell migration assay (Figure S3C) revealed that, following poly(dA: dT) stimulation, HAQ cells displayed a reduced ability to recruit T cells (Jurkat-CXCR3) and NK cells (NK92) to tumors in comparison to WT cells (Figure 3I). Finally, we introduced *STING1* cDNA variants, WT or HAQ, into cancer cell lines where the endogenous *STING1* had been completely knocked out using CRISPR (Figure 3J). cGAMP treatment revealed a significant reduction in IFN-β upregulation, as detected by ELISA, in cells expressing HAQ cDNA compared to WT (Figure 3K). These *STING1* cDNA variants were also transduced into 293-Dual Null reporter cells (Figure 3L), which lacked endogenous STING1 expression^66^ and contained dual reporters (Figure 3L). Consistently, cells expressing HAQ exhibited reduced IRF binding and IFN-β upregulation following cGAMP stimulation compared to cells expressing WT (Figure 3M). Taken together, our results demonstrate that the *STING1* variant HAQ exhibits a weaker response to cGAMP stimulation in comparison to the WT, leading to a reduction in IFN-I production and lower expression of ISGs in cancer cells.

### *STING1* variants are associated with diverse tumor immunity and treatment responses targeting the intrinsic IFN-I signal

**Figure 4.**
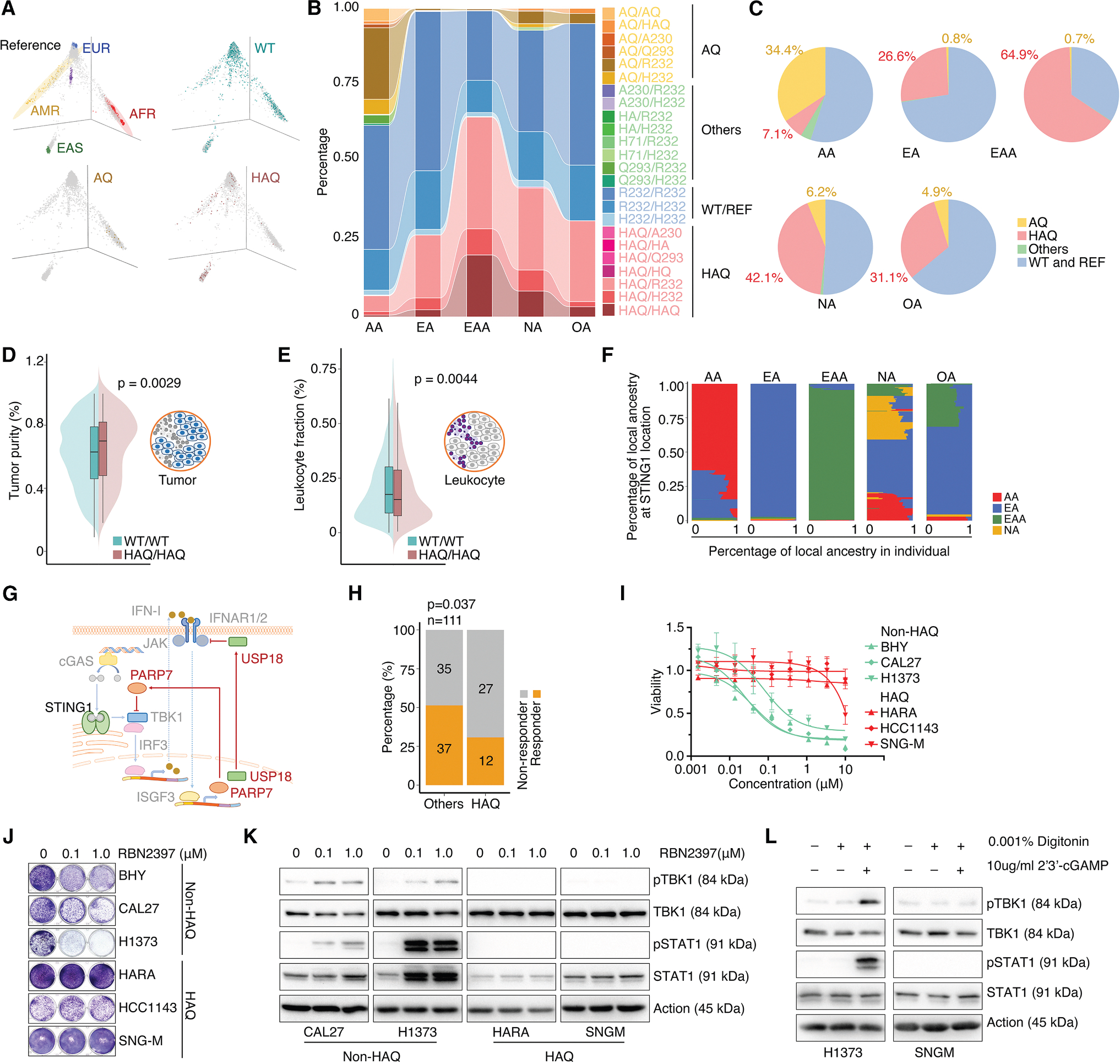
*STING1* variants are associated with diverse tumor immunity and treatment responses targeting the intrinsic IFN-I signal (A) Three-dimensional visualization of genetic variation in individuals from the reference populations and TCGA patients on the first three principal components (PCs) generated by EIGENSTRAT. In the top left, colors represent reference populations, while gray represents TCGA patients. The other graphs display individuals from TCGA with the *STING1* WT/WT (green), AQ/AQ (orange), and HAQ/HAQ (dark red) haplotypes. (B) Alluvial plot illustrates the distribution of distinct *STING1* haplotypes among five genetic ancestry populations in TCGA cohort. (C) Pie plots illustrate the distribution of four major *STING1* haplotype groups among five genetic ancestry populations in TCGA cohort. Colors represent the different haplotype groups for the *STING1* gene. (D) Comparison of the tumor purity in TCGA specimens harboring *STING1* WT/WT or HAQ/HAQ haplotypes, with statistical significance determined by a meta *p* value after adjusting for genetic ancestry and cancer type. (E) Comparison of the leukocyte fraction in TCGA specimens harboring *STING1* WT/WT or HAQ/HAQ haplotypes, with statistical significance determined by a meta *p* value after adjusting for genetic ancestry and cancer type. (F) Distribution of local ancestry at the *STING1* genomic locus in TCGA patients. For each individual, local ancestry was normalized by their global ancestry, inferred through STRUCTURE. Each color corresponds to one of the four reference ancestries used in the local ancestry estimation. TCGA patients were grouped into five ancestry categories based on EIGENSTRAT, represented as five bars. Within each ancestry group, patients are shown as rows segmented by colors reflecting their genetic ancestry composition. The rows are ordered by hierarchical clustering using Ward’s method, with the distance matrix derived from the cosine dissimilarity of genetic composition. (G) An illustration of the IFN-I pathway and its feedback regulation loops mediated by PARP7 and USP18. (H) The percentage of PARP7i-sensitive and -resistant cell lines in cells with *STING1* non-HAQ and HAQ haplotypes. (I) The viabilities of cancer cells following a 72-h treatment with PARP7i (RBN2397) were evaluated using MTT assays in six cancer cell lines with different *STING1* haplotypes. (J) A representative image of the colony formation assay for cells treated with RBN2397 for 6 days in six cancer cell lines with different *STING1* haplotypes. (K) Western blots were used to detect TBK1 and STAT1 phosphorylation after 48 h of RBN2397 treatment in cell lines with different *STING1* haplotypes. (L) Western blots were used to detect TBK1 and STAT1 phosphorylation after 30 min of cGAMP treatment in cell lines with different *STING1* haplotypes.

To investigate nsSNPs in the *STING1* gene in patients with cancer, we estimated the genetic ancestry of TCGA patients using the EIGENSTRAT algorithm.^67^ Reference eigenvectors were established using SNP information from the 1000 Genomes Project cohort, which were then applied to determine the genetic ancestries of each TCGA patient (Figure 4A). Consistent with the distribution among geographically structured human populations from the 1000 Genomes Project, we observed that TCGA patients in the US population displayed a diverse pattern of nsSNPs in the *STING1* gene among the patients from different genetic ancestries (Figure 4B). For instance, the HAQ haplotype is primarily observed in East Asian American (EAA) populations, whereas the AQ haplotype is more prevalent within AA populations (Figure 4C). Leveraging the substantial sample size of the TCGA cohort, which allows us to select homogeneous haplotypes such as WT/WT and HAQ/HAQ, we performed an analysis of the correlation between haplotype and phenotype. Given that the STING-mediated intrinsic innate immune signal may influence tumor immunity and inflammation, we analyzed the ratio of tumor cells to infiltrating stromal cells, mainly immune cells, across the TCGA cohort. At the pan-cancer level, patients with the WT/WT haplotype exhibited significantly lower tumor purity levels compared to those with the HAQ/HAQ haplotype, with statistical significance determined by a meta *p* value (adjusted for genetic ancestry and cancer type, meta *p* = 0.0029, Figure 4D; adjusted for genetic ancestry and cancer subtype, meta *p* = 0.0187). Consistent with this, patients with the WT/WT haplotype showed significantly higher levels of tumor-infiltrating leukocytes when compared to patients with HAQ/HAQ, with statistical significance determined by a meta *p* value (adjusted for genetic ancestry and cancer type, meta *p* = 0.0044, Figure 4E; adjusted for genetic ancestry and cancer subtype, meta *p* = 0.0095). We employed CIBERSORT to estimate the proportions of different immune cell types within the tumor microenvironment.^68^ Although the per-cancer-type sample sizes for homozygous *STING1* genotypes were insufficient for robust statistical testing, patients with the WT/WT haplotype showed a trend toward globally higher leukocyte infiltration compared with those with HAQ/HAQ (Figure S5A). As expected, the composition of infiltrating immune cells varied markedly across cancer types, suggesting that the impact of the *STING1* haplotype on immune infiltration is cancer-type dependent. Considering that a significant portion of the US population is admixed (Figure 4A), we employed LAMP to quantitatively assess the ancestral composition for each patient.^69^ A substantial percentage of the TCGA specimens displayed a blend of ancestral influences in the *STING1* region from multiple distinct populations (Table S12). The AA population exhibited higher levels of combined local ancestry with European ancestry, whereas the EA population showed the lowest levels of mixed ancestry in the *STING1* region (Figures 4F and S5B). This result suggests that future functional and translational studies of STING1 should carefully account for both global and local ancestry context to fully capture the impact of genetic diversity at this locus.

Targeting genes in the intrinsic IFN-I pathway of cancer cells has been proposed as a therapeutic strategy in oncology.^51,70^ Given that *STING1* haplotypes are associated with differential tumor-derived IFN-I activity, which regulates not only antitumor immunity but also cancer cell fitness,^51,70^ we hypothesized that *STING1* variants may be associated with differing responses to the perturbation of genes in the IFN-I pathway in cancer cells. Therefore, we analyzed the correlation between *STING1* haplotypes and dependencies of 198 genes in the IFN pathway. Cell growth dependency of each gene in 886 non-hematological cancer cell lines was estimated based on the CRISPR-Cas9 screen profiles generated by the DepMap project.^71^ Six genes in the IFN-I pathway show trends in dependency differences between cell lines with different *STING1* haplotypes, specifically WT versus HAQ (Figure S5C). Interestingly, two IFN-I pathway repressors, PARP7 and USP18, which negatively regulate IFN activity by inhibiting TBK1 and JAK1 functions, respectively (Figure 4G), exhibited the strongest association in cell dependency among these genes (Figure S5C). Supporting their classification as ISGs, the expression of PARP7 and USP18 showed a significant positive correlation with the ISG signature across cancer cell lines (Figures S5D and S5E). In a hypothetical scenario, in WT cells, heightened intrinsic IFN-I activity triggers the upregulation of repressive feedback mechanisms involving PARP7 and USP18, ultimately inhibiting IFN-I and promoting cell tolerance. Therefore, the removal of these repressors could increase IFN-I levels, leading to cell death.^51,70^ In HAQ cells with lower IFN-I activity, the significance of feedback repression is diminished, making them less susceptible to the effects of PARP7 and USP18 knockout. To functionally validate this genetic observation, we retrieved the chemical PARP7 inhibitor (PARP7i) response profiles of 111 cancer cell lines with *STING1* haplotype information.^70^ Based on the response profiles of three different PARP7i treatments, the cancer cell lines were divided into sensitive and resistant groups using K-means cluster analysis.^70^ Consistently, we observed that cancer cells carrying HAQ exhibited a significantly higher proportion of cells resistant to PARP7i treatment compared to cancer cells lacking HAQ (*p* = 0.037; Figure 4H). This result was further experimentally validated by MTT (Figures 4I and S4B) and colony-formation assays (Figures 4J and S4C) in six cancer cell lines by treatment with a PARP7i (RBN-2397) that is currently being evaluated in a phase 1 clinical trial.^72^ Western blots revealed that, following RBN-2397 treatment, there was a remarkable upregulation in the phosphorylation of TBK1 and STAT1 in WT cells, indicating that PARP7 inhibition enhanced intrinsic IFN-I activity. In contrast, lower or no phosphorylation of these proteins was observed in HAQ cells (Figures 4K and S4D). Consistently, these HAQ cells showed a weaker response to the direct activation of STING1 by stimulation of cGAMP compared to WT cells (Figures 4L and S4E). Taken together, our observations indicate that, beyond influencing ICI treatment, *STING1* haplotype may also affect small-molecule targeted therapy in cancer, specifically targeting the intrinsic IFN-I pathway in cancer cells.

### Genome-wide characterization of immune-related genes with significant nsSNP diversity across genetic ancestry populations

**Figure 5.**
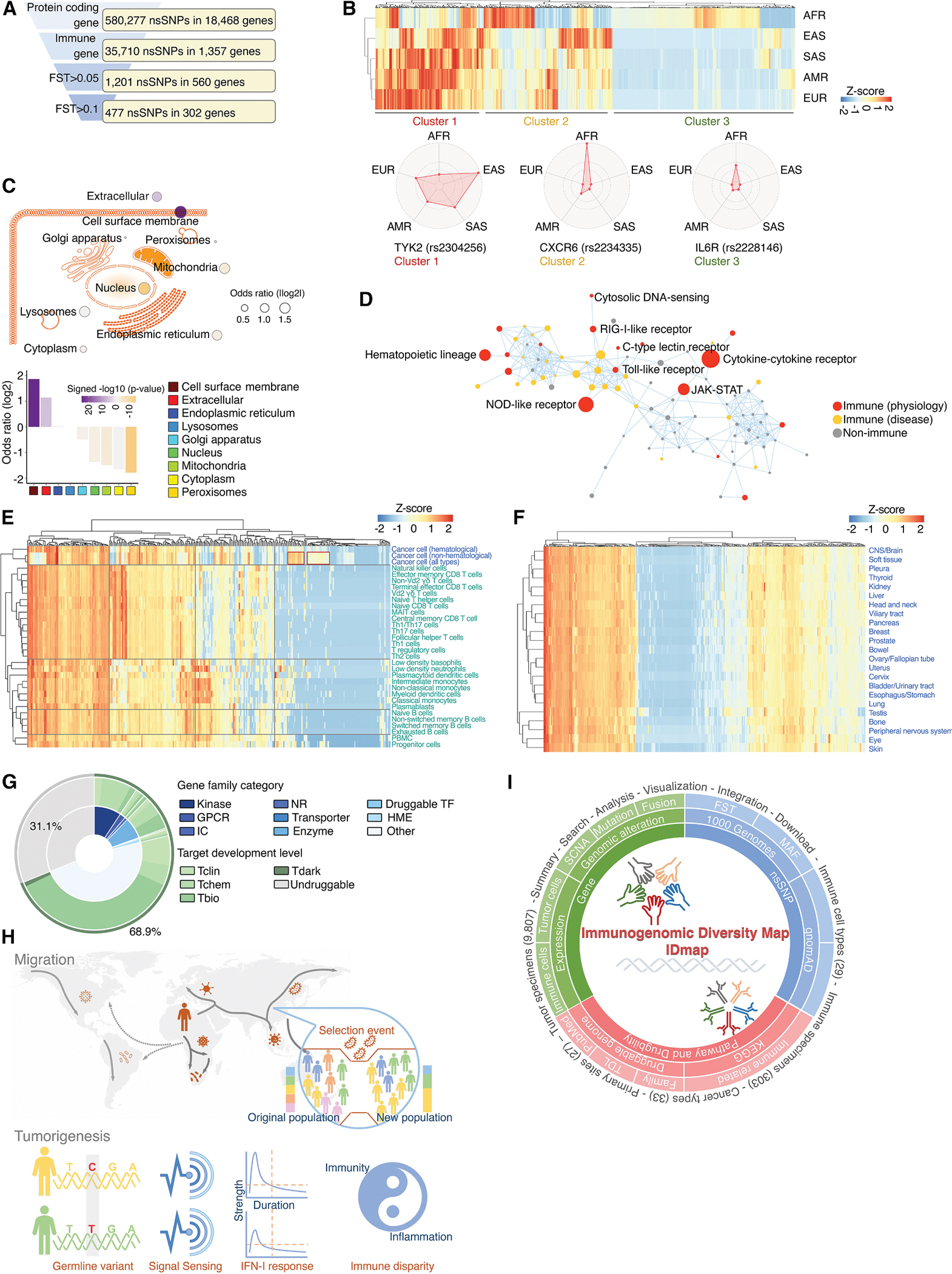
Genome-wide characterization of immune-related genes with significant nsSNP diversity across genetic ancestry populations (A) Workflow for identifying nsSNPs within immune-related genes with significantly different frequencies among the major geographically structured populations. (B) Top: unsupervised cluster analysis of minor allele frequency (MAF) of the nsSNPs within IDgenes across major geographically structured populations. Three distinct clusters were identified based on the frequency and distribution of these nsSNPs among populations. Bottom: radar plots illustrate the MAF of representative nsSNPs within each IDgene cluster across major geographically structured populations. (C) The bubble plot (top) and bar plot (bottom) show the enrichment of proteins encoded by IDgenes in corresponding subgroups based on their subcellular locations. Top: all proteins were grouped based on their subcellular locations, and the enrichment of proteins encoded by IDgenes in each group is represented by a bubble plot. Bottom: a bar plot summarizes the enrichment of proteins encoded by IDgenes in each group. *p* values were calculated using Fisher’s exact test. Purple indicates enrichment; green indicates depletion. Bubble size represents the absolute value of log2(OR). (D) Network connections of the IDgene-enriched KEGG pathways. Circle diameter represents fold enrichment, with immune-associated routes highlighted. Connecting lines indicate shared genes among pathways. (E) Unsupervised cluster analysis of expression level of IDgenes across DepMap cancer cell lines and 29 immune cell populations. Each row represents a cell type, and each column corresponds to an individual IDgene. (F) Unsupervised cluster analysis of expression levels of IDgenes in 23 non-hematological cancers from DepMap. Each column represents an individual gene, and each row corresponds to a specific cancer type. (G) Classification of IDgenes based on druggability (inner circle) and target development level (TDL; outer circle). (H) Hypothesis of IDgenes. (I) Overview of the Immunogenomic Diversity Map (IDmap) database portal.

Our above proof-of-concept study indicates that the diversity of nsSNPs within immune-related genes could influence cancer immunity among human populations. To systematically characterize these nsSNPs at a genome-wide scale, we employed F_ST_ thresholds of >0.1 and >0.05, identifying 477 (tier 1) and 1,201 (tier 2) nsSNPs within 302 and 560 immune-related genes, respectively (Figure 5A; Table S13). Here, we termed the immune-related genes that were annotated by KEGG with these diverse nsSNPs as immunogenomic diversity genes (IDgenes), i.e., the genes that functionally contribute to immunity and exhibit population-diverse nsSNPs. Tier 1 nsSNPs within IDgenes were further categorized based on their distribution and frequencies among populations into three clusters (Figure 5B). These clusters may reflect distinct selection pressures and provide a framework for future mechanistic studies on population-level immune diversity and cancer susceptibility. The notable depletion (e.g., cluster 1) or enrichment (e.g., clusters 2 and 3) of these nsSNPs in certain populations suggests that investigating their functional impact could provide valuable insights into the diversity of immune responses across populations.

When classifying all proteins based on their subcellular localization, we found that IDgene-encoded proteins were significantly enriched in two groups: extracellular and surface proteins (*p* = 1.37e–6 and *p* = 9.72e–26, respectively; Figure 5C). Consistently, Gene Ontology (GO) analysis revealed a significant enrichment of these genes in the cytokine-cytokine receptor interaction pathway (Figure 5D; Table S14). As expected, IDgenes were overrepresented in immune-related pathways, particularly those associated with innate immunity, such as PRR signaling pathways. Interestingly, some IDgenes also participated in non-immune pathways beyond their immune functions (Figure 5D), suggesting a complex interplay between immune and non-immune signaling networks. The next question is whether these immune-related IDgenes are intrinsically expressed in tumor cells in addition to their expression in immune cells. To address this, we integrated RNA-seq profiles from DepMap cancer cell lines and 29 immune cell types within the peripheral blood mononuclear cell fraction of healthy donors.^73^ As anticipated, many IDgenes displayed lineage-restricted expression patterns within immune cells (Figure 5E). However, approximately half (47%, 142/302) were reliably detected across cancer cell lines, defined as fragments per kilobase million (FPKM) >1 in more than 50% of cell lines. After exclusion of hematopoietic malignancies, a subset of IDgenes demonstrated consistent expression across the majority of solid cancer types (Figure 5F), suggesting their involvement in conserved immune defense mechanisms intrinsic to the diverse normal tissues from which these tumors originate. In contrast, another subset of IDgenes displayed lineage-enriched expression patterns across non-hematopoietic cancer cell lines, implying more lineage-specific functional roles. Finally, given that the modulation of immune-related genes by small-molecule inhibitors has been extensively investigated in oncology, we assessed the druggability of these IDgenes using a predictive strategy based on sequence and structural similarities within conserved protein domains.^48^ Druggability analysis revealed that approximately 68.9% of IDgenes (*n* = 208) were classified as druggable targets (Figure 5G). Many of them belong to well-characterized druggable protein families, such as kinases. Consistently, a significant proportion of these druggable IDgenes are annotated with a high target development level (TDL), including Tclin (14.9% [31/208], representing targets with at least one approved drug in clinical use) and Tchem (23.1% [48/208], representing targets known to bind small molecules with high confidence), by the Pharos database.^74^ For instance, inhibitors targeting JAK have received FDA approval for cancer treatment,^75^ and STING1 agonists are currently under evaluation in clinical trials.^76–78^

Collectively, our results have identified a group of nsSNPs within immune-related genes that exhibit notably distinct distributions across different genetic ancestry populations. This divergence likely arises from differing selection pressures, such as historical pathogen exposure, experienced during human evolution and migration (Figure 5H). Although some of these nsSNPs have been partially characterized for their biological significance, many remain underexplored. Nonetheless, they hold potential as part of the genetic foundation for understanding disparities in immunity and associated diseases, such as cancer. To facilitate further investigation into their functions, we have created a publicly accessible database called the Immunogenomic Diversity Map (IDmap; Figure 5I). It provides visualization for metrics such as F_ST_ values, allele frequency across human populations, gene expression profiles, genomic alterations, and pathway annotations.

## DISCUSSION

The immune system has undergone substantial evolution as a result of pathogen-driven selection pressures throughout human history.^1–5^ We observed significantly higher F_ST_ values in nsSNPs within immune-related genes across major geographically structured human populations at a genome-wide scale, indicating substantial genetic variation in immune pathways. In contrast, cancer-related genes exhibit lower F_ST_ values, suggesting less diversity among populations. The essential cellular functions performed by cancer-related genes may subject them to strong purifying selection pressure. Consistent with previous observations,^79^ we detected signatures of both strong positive selection, favoring variants that provide advantages in specific environments, and purifying selection (negative selection), which eliminates harmful genetic changes, within immune-related genes. Furthermore, we observed a negative correlation between F_ST_ values and recurrent genomic scores, indicating that genes involved in cancer-driving events have less diversity across human populations. As expected, immune-related genes exhibit lower recurrent genomic scores, suggesting that, during tumorigenesis, they serve in permissive roles rather than driving events.

Identification of functional nsSNPs in cancer immunity is challenging due to the complexity of tumor-immune interactions and the fact that common nsSNPs typically have subtle effects. Although the extensive collection of human cancer cell lines with well-characterized genotyping profiles from the DepMap project provides a unique and simplified model for concept-proving studies, it still lacks the statistical power required to detect genome-wide germline variants in trans, as expected in most classical population genetic studies. To address this limitation, we employed a multi-factor ranking strategy to prioritize candidate nsSNPs based on their biological relevance. By leveraging the intrinsic IFN-I signature estimated from RNA-seq profiles of cancer cells, we identified a set of nsSNPs that are widely distributed across various genetic ancestral populations and have the potential to influence anti-tumor innate immunity. STING1 acts as a central protein in cancer cells, sensing both endogenous and treatment-induced cytoplasmic DNA. It plays a pivotal role in regulating antitumor immune responses, primarily through tumor-intrinsic signals such as IFN-I.^76–78^ A recent GWAS utilizing the TCGA cohort identified the H232 allele in *STING1* as being associated with decreased IFN signaling across cancers.^41^ This independent discovery and our findings provide robust evidence for the functional significance of nsSNPs in *STING1* within the context of IFN pathway activity in human cancers. The diverse distribution of less-responsive variants (HAQ) of *STING1* within the EAA population strongly suggests that genetically mediated disparities in tumor immunity may exist. Notably, beyond HAQ, additional *STING1* variants such as R232H, R293Q, and AQ also display biologically meaningful effects.

Tumor-immune interactions are highly intricate processes characterized by dynamic interplay between cancer cells and the immune system. This complexity makes it challenging to elucidate the functions of genetic variants involved in tumor immunity compared to studying those variants in cancer pathways. Although we have demonstrated that *STING1* variants influence intrinsic IFN-I activity in cancer cells in response to cGAS-mediated cyclic dinucleotide (CDN) signals, we lack knowledge regarding how they impact immune cell functions within the context of tumorigenesis. Excitingly, HAQ knockin mouse models have been developed to explore the physiological function of the HAQ variant *in vivo*,^61,80^ providing a valuable model for defining the role of *STING1* variants in tumor immunity. Most immune-related genes have functions that extend beyond immunity, adding an additional layer of complexity. For example, apart from its role in innate immune responses, STING1 also plays crucial roles in autophagy and DNA damage responses.^76–78^ Therefore, understanding how these nsSNPs function in non-immune mechanisms and their connection with their immune functions is another important but unaddressed question. The functions of common nsSNPs confer weak effects, often insufficient to elicit strong phenotypic changes, adding another layer of challenge when investigating common genetic variations in comparison to pathogenic rare variants. Considering that the cumulative effects of multiple common nsSNPs in immune-related genes may be associated with more substantial impacts on tumor immunity, polygenic scores could be a promising future solution to capture the collective function of these nsSNPs.

Our findings suggest that genetic diversity in immune-related genes may contribute to disparities in tumor immunity, inflammation, and, potentially, the efficacy of immunotherapy across populations with diverse genetic ancestries. Furthermore, as increasing evidence suggests that tumor immunity also influences outcomes in targeted therapies and chemotherapy, this genetic diversity could also be linked to varying responses to non-immunotherapy treatments in cancer. Thus, given the various distributions of these nsSNPs among human populations, it is crucial to include a sufficient number of minority patients in clinical trials. Our findings also support the concern whether clinical trials of immune checkpoint inhibitors conducted on a monolithic population could necessarily be translated to more genetically diverse populations.^81,82^

### Limitations of the study

Although we identified correlations between PRR pathway nsSNPs and intrinsic IFN-I activity, the number of available cancer cell lines is still relatively small, limiting statistical power for genome-wide discovery. Likewise, while TCGA represents the largest cancer genomics dataset, the representation of minority populations such as AA and Asian American patients remains insufficient, reducing our ability to fully assess ancestry-specific effects. In addition, the pharmacogenetic analysis of PARP7i response was based on 111 cancer cell lines, which, although the largest dataset currently available, remain modest for detecting subtle genotype-drug associations. Despite these limitations, we performed additional genetic and functional validation, including the use of isogenic cell models and pharmacologic perturbations, which consistently supported the correlation between *STING1* variants, intrinsic IFN-I signaling, and therapeutic response. These complementary approaches strengthen our confidence in the observations. Finally, our study indicates a broader need in the field: the generation of larger and more diverse cancer cell line collections and clinical cohorts that better represent distinct human populations will be critical for advancing the understanding of germline variation in cancer immunity and therapy response.

## RESOURCE AVAILABILITY

### Lead contact

Requests for further information and resources should be directed to and will be fulfilled by the lead contact, Dr. Xiaowen Hu (xiaowenh@upenn.edu).

### Materials availability

This study did not generate new, unique reagents.

### Data and code availability

- This paper analyzes existing, publicly available data. Information about the 1000 Genomes phase 3 can be found at the 1000 Genomes Project website (https://www.internationalgenome.org/data-portal/data-collection/grch38). Information about TCGA and the TCGA research network can be found at the TCGA project website (http://cancergenome.nih.gov). The genomic profiles in a large-scale cancer cell line panel are publicly available through the Dependency Map (DepMap) portal (https://depmap.org/portal/) and the Score projects (https://doi.org/10.6084/m9.figshare.c.5289226.v1).
- The database created by this study is publicly available through the IDmap data portal (http://52.25.87.215/IDmap/).
- RNA-seq data generated in this work are deposited in the GEO database under accession number GEO: GSE314074.
- This paper does not report original code.
- Any additional information required to reanalyze the data reported in this paper will be made available by the lead contact upon reasonable request.

## STAR★METHODS

### EXPERIMENTAL MODEL AND STUDY PARTICIPANT DETAILS

#### Cell lines and culture conditions

Cell lines were purchased from the ATCC, DSMZ, JCRB, NCI Development Therapeutics Program, or Invivogene, with detailed information and culture medium listed as following. All cell lines were tested negative for mycoplasma contamination. 293T (RRID: CVCL_0063), 293-Dual^™^ Null Cells, BHY (RRID:CVCL_1086), and CAL 27 were cultured in DMEM medium (Invitrogen) supplemented with 10% fetal bovine serum (Sigma). NCI-H1373 (RRID:CVCL_1465), HARA ((RRID:CVCL_2914), HCC1143 (RRID: CVCL_1245), SK-OV-3 (RRID:CVCL_0532), TOV-21G, and Jurkat were cultured in RPMI1640 medium (Invitrogen) supplemented with 10% fetal bovine serum. SNG-M was cultured in F-12 medium (Invitrogen) supplemented with 20% fetal bovine serum. NK-92 was cultured in MyeloCult^™^ H5100 (STEMCELL) supplemented with 100 units/ml IL2 (STEMCELL). All other cells were cultured in RPMI1640 medium (Invitrogen) supplemented with 10% fetal bovine serum. All cell lines were maintained at 37°C and 5% CO2. Cell lines were obtained from the indicated vendors and authenticated by the suppliers prior to distribution. All experiments were performed using early-passage cells to avoid genetic drift and phenotypic variation. Cell cultures were routinely monitored for mycoplasma contamination using the MycoAlert Mycoplasma Detection Kit (Lonza) according to the manufacturer’s instructions.

#### Human sample information

Publicly available, de-identified genomic and clinical datasets from the 1000 Genomes Project and The Cancer Genome Atlas (TCGA) were used in this study. The 1000 Genomes Project provides population-scale human genetic variation data derived from healthy individuals across diverse ancestries. TCGA provides comprehensive molecular profiling data from human tumor and matched normal samples, together with associated clinical annotations, including age, biological sex, tumor type, and clinical outcome, where available. Detailed information on sample acquisition, inclusion criteria, ethical approvals, informed consent procedures, and data processing pipelines has been previously reported by the respective consortia and is publicly accessible through their primary publications and data portals. Biological sex information, when available, was obtained directly from TCGA metadata and incorporated into downstream analyses, as appropriate.

### METHOD DETAILS

#### Identification of nsSNP in 1000 genome data

Variant call data for all chromosomes of the 1000 Genomes Phase 3 ^44^ were downloaded from the data portal (https://www.internationalgenome.org/data-portal/data-collection/grch38). Only single nucleotide variants (SNVs) were retained for downstream analysis. The SNV call data were functionally annotated using SnpEff ^85^ (https://pcingola.github.io/SnpEff/), and non-synonymous SNPs were identified. Only autosomal nsSNPs were included in our study.

#### Estimates of genetic differentiation

Genetic differentiation for both autosomal protein-coding genes and nsSNP loci was measured using one of Wright’s F statistics, the fixation index Fst ^46,98^. The Fst value quantifies the proportion of genetic variance based on allele frequencies ^99^ and is calculated as follows:

$$Fst=\frac{H_{T}\;-\;HS}{HT}$$

where HT represents the variation between populations and HS represents the variation within populations.

By comparing the average heterozygosity within a subpopulation to the average heterozygosity within the metapopulation, we could estimate the genetic differentiation within and among populations. The R package ‘popgenome’ ^86^ was used to estimate both global Fst and pairwise F_ST_ values between subpopulations. VCF files from the 1000 Genomes Phase 3 for 22 autosomes were used as input. The genome-wide data were then split into genes based on genomic positions using the ‘splitting.data()’ function. Global F_ST_ was calculated using the ‘F_ST.stats()’ function in the mode of “nucleotide”. Pairwise F_ST_ values were extracted using ‘nuc.F_ST.pairwise’. Site-specific F_ST_ values were obtained by setting ‘subsites = nonsyn, site.FST=True’.

#### Estimates of natural selection for protein coding genes by McDonald–Kreitman test

The natural selection of protein-coding genes was estimated using the McDonald–Kreitman (MK) test ^50^ by inferring the proportion of positive selection (α) ^100^ and Neutrality index (NI) ^101^. The MK test compares the ratio of nonsynonymous to synonymous substitutions within a species (polymorphism) to the ratio of nonsynonymous to synonymous fixed differences between this species and its closely related species (divergence). Here, we use chimpanzee as the outgroup to calculate the divergence. Nl and α values were determined as follows:

$$Nl=\frac{Pn/Ps}{Dn/Ds}$$

$$\alpha =1-\frac{PnDs}{PsDn}$$

where Pn is the number of nonsynonymous variants within the species, Ps is the number of synonymous variants within the species; Dn and Ds represent the numbers of nonsynonymous and synonymous variants between species, respectively. An NI value greater than 1 indicates purifying selection in effect, while an NI value less than 1 indicates positive selection. A positive α value suggests positive selection, whereas α<0 suggests negative selection. To calculate NI and α values, we retrieved human variants from the 1000 Genomes Project Phase 3 GRCh38, which included 2548 samples. The human-chimpanzee alignment and variant calling file were downloaded from Prado-Martinez J, et al. ^102^, which provides variant data from 25 chimpanzee samples aligned to the human reference genome (hg18). To ensure compatibility with our analysis pipeline based on the GRCh38 human genome build, we performed a liftover of the chimpanzee variant coordinates from hg18 to GRCh38 using the Picard ‘LiftoverVcf’ tool (https://broadinstitute.github.io/picard/) with the appropriate UCSC chain file. The VCF files of human 1000 genome and chimpanzee were merged and submitted to the R package ‘popgenome’ for the estimation of NI and α values for each protein-coding gene using the function ‘MKT’.

#### Processing of TCGA genomic profiles

Genomic profiles from The Cancer Genome Atlas (TCGA), encompassing copy number alterations, mutations, and fusions, were analyzed using a standardized pipeline previously developed by our team ^48,49^. Recurrent somatic copy number alterations (SCNAs), mutations, and fusions were identified for each patient following the methodologies outlined in our prior work and further described below.

#### RNA-seq data processing

The poly(A)^+^ RNA-seq data for primary tumors, their adjacent tissues, and normal tissue from GTEx project were generated by the University of North Carolina and the British Columbia Cancer Agency Genome Sciences Centre as part of the TCGA project. The poly (A)^+^ RNA-seq data for hematopoietic cells were download from Sequence Read Archive (SRA, accession number SRP125125), and the poly(A)^+^ RNA-seq data for lymphatic tissues were download from the Human Protein Atlas (HPA), Illumina’s Human BodyMap 2.0 project, and Encyclopedia of DNA Elements (ENCODE). All RNA-seq data were processed through a pipeline developed by the UCSC Toil RNAseq Recompute Compendium, which allowed us to consistently process large-scale RNA-seq data without computational batch effects ^103,104^. For TCGA RNA-seq data, if more than one sample existed for a participant, one single tumor sample (and matched adjacent sample, if applicable) was selected based on the following rules: (1) tumor sample type: primary (01) > recurrent (02) > metastatic (06); (2) order of sample portions: higher portion numbers were selected; and (3) order of plate: higher plate numbers were selected.

#### SNP array data collection and processing

Single-nucleotide polymorphism (SNP) array data (Affymetrix Genome-Wide Human SNP Array 6.0) in CEL format across 33 cancer types were retrieved from the TCGA Data Portal (https://tcga-data.nci.nih.gov/tcga/). Segmentation files of TCGA tumor samples processed by circular binary segmentation (CBS) algorithm ^105^ were retrieved from the TCGA GDAC Firehose of the Broad Institute (http://gdac.broadinstitute.org/; retrieval date: Jan, 3, 2018). If multiple samples existed for a participant, one pair of tumor and matched control was selected for ABSOLUTE analysis and one tumor sample was kept for focal SCNA analysis. Sample selection was based on the following rules: (1) sample type: for tumor tissues, primary (01) > recurrent (02) > metastatic (06); for normal control tissues, blood (10) > solid (11); (2) molecular type of analyte for analysis: preference for D analytes (native DNA) over G, W, or X (whole-genome amplified); (3) order of sample portions: higher portion numbers were selected; and (4) order of plate: higher plate numbers were selected.

#### Recurrent focal SCNA estimation

The Genomic Identification of Significant Targets in Cancer (GISTIC 2.0) algorithm ^87^ (https://www.broadinstitute.org/cancer/cga/gistic) was used to identify significantly recurrent focal genomic regions that were gained or lost in a given tumor type. Segmentation files retrieved from the TCGA GDAC Firehose of the Broad Institute were used as input. GISTIC deconstructed copy number alterations into broad and focal events and applied a probabilistic framework to identify location and significance levels of SCNAs. For recurrent focal SCNA estimation, the significance levels (q values) were calculated by comparing the observed gains/losses at each locus to those obtained by randomly permuting the events along the genome. Tumors which had more than 2,000 segments were excluded from our analysis. Default parameters of GISTIC were used with the confidence level set to 0.99 (by -conf). Focal events with q-value below 0.25 were considered as significantly recurrent. Significant focal events in individual samples were then classified into four categories according to the amplitude threshold of GISTIC: GISTIC status=0, below threshold; GISTIC status=1, amplified (gain); GISTIC status=2, highly amplified (amplification); GISTIC status =−1, deleted (loss); GISTIC status=−2, highly deleted (deletion). In each cancer type, a GISTIC score (G-score), which accounts for both frequency and amplitude of a given SCNA event ^87^, was generated by GISTIC for each gene and separately for gain or loss. Genes with a G-score < 0.1 were excluded from downstream analysis due to low frequency and/or amplitude. For a given gene, an overall G-score across all cancer types was calculated by an unweighted sum of G-scores in every cancer type.

#### Correlation analysis between copy number and RNA expression

To identify genes that had positive correlations between their RNA expression levels and copy number alterations, the putative gene-level copy number of a given gene was estimated by the GISTIC algorithm. Genes that were detectable in at least 10% of tumor specimens (90th percentile of FPKM value ≥1) in a given cancer type were subjected to correlation analysis. Pearson correlation analysis was performed by R software and the threshold of significant correlation between the estimated copy number and RNA expression level for each gene was set to p<0.001 (Pearson’s correlation).

#### Recurrent SCNA score estimation

At the individual cancer type level, we identified putative cancer-associated genes driven by SCNAs using four criteria: 1) location in a peak region of a significantly recurrent focal SNCA locus estimated by GISTIC (q≤0.25); 2) alteration with high frequency and large amplitude (G-score ≥0.1); 3) mRNA expression reliably detected in at least 10% of tumor specimens in a given cancer type (the 90^th^ percentile of FPKM value ≥1); and 4) expression level of mRNA significantly and positively correlated with the estimated copy numbers (p value of Pearson’s correlation coefficient between log[FKPM+0.001] and log ratio < 0.001). To estimate SCNAs for these putative cancer-associated genes at a pan-cancer level, we calculated an overall G-score by an unweighted numeric sum of G-scores that met all four criteria in each individual cancer type.

#### Whole-exome sequencing data collection and processing

Mutation Annotation Format (MAF) profiles for 33 cancer type were downloaded from the TCGA Multi-Center Mutation Calling in Multiple Cancers (MC3) project (https://doi.org/10.7303/syn7214402), a variant calling project of TCGA ^106^. The MC3 data was generated through seven independent mutation calling algorithms, including Pindel (INDEL), MuSE (SNV), Radia (SNV) ^107^, VarScan2 (SNV/INDEL), MuTect (SNV), Indelocator (INDEL) and SomaticSniper (SNV). Variants from each caller were merged, QC filtered and stored in MAF file ^106^. If multiple samples existed for a participant in the MAF, one single pair of tumor/matched control sample was kept following these rules: (1) sample type: for tumor tissues, primary (01) > recurrent (02) > metastatic (06); for normal tissues, blood (10) > solid (11); (2) molecular type of analyte for analysis: preference for D analytes (native DNA) over G, W, or X (whole-genome amplified); (3) order of sample portions: higher portion numbers were selected; and (4) order of plate: higher plate numbers were selected. We excluded all mutations that were not tagged with PASS or WGA alone in all cancer types.

#### Recurrent mutation estimation

To predict the putative cancer-associated genes driven by mutation, five independent methods were integrated and applied to identify recurrent mutations: (1) MutSigCV (http://software.broadinstitute.org/cancer/software/genepattern/modules/docs/MutSigCV), which identifies genes that are significantly mutated in cancer genomes, using a model with mutational covariates. It analyzes the mutations of each gene to identify genes that were mutated more often than expected by chance, given the background model; (2) Oncodrivefm (http://bg.upf.edu/group/projects/oncodrive-fm.php), which computes a metric of functional impact using three well-known methods (SIFT, PolyPhen2 and MutationAssessor) and assesses how the functional impact of variants found in a gene across several tumor samples deviates from a null distribution to detect candidate driver genes; (3) OncodriveCLUST (http://bg.upf.edu/group/projects/oncodrive-clust.php), which is designed to exploit the feature that mutations in cancer genes, especially oncogenes, often cluster in particular positions of the protein and change their functions; thus, this feature can be used to nominate novel candidate driver genes; (4) ActiveDriver (http://reimandlab.org/software/activedriver/), which identifies post-translational modification (PTM) sites in proteins (i.e., active sites such as signaling sites, protein domains, regulatory motifs) that are significantly mutated in cancer genomes; and (5) HotSpot3D (https://github.com/ding-lab/hotspot3d), which identifies mutation hotspots from linear protein sequence and correlates the hotspots with known or potentially interacting domains and mutations. MC3 MAF files excluding hypermutated samples were used as input for the above programs, and default parameters were used for all five programs. A mutation index x (ranging from 0 to 5) was assigned to genes which passed the threshold of x out of five programs for a given cancer type. In addition, a mutation score (M-score) was calculated for each mutated gene in a given cancer type, which takes into account both the mutation index and frequency of mutation across samples (i.e., M score = mutation index × mutation frequency). Genes with mutation index ≥ 2 (identified as positive by at least two programs) were considered to be recurrently mutated. An overall M-score was generated to measure the recurrent mutation level of a given gene across all cancers, by unweighted sum of M-scores estimated for each individual cancer type.

#### Transcript fusion data collection and analysis

The gene fusion data of TCGA were retrieved from TumorFusions data portal (http://tumorfusions.org/), which analyzed transcript fusions across 33 cancer types from TCGA ^108^. Transcript fusion events were called by Pipeline for RNAseq Data Analysis (PRADA) ^109^, and fusions detected in normal samples were excluded. Six filters controlling for sequence similarity of the partner genes, transcriptional allelic fraction, dubious junctions, germline events and presence in non-neoplastic tissue were applied ^108^. If more than one sample existed for a participant, one single sample was kept following these rules: (1) sample type: for tumor tissues, primary (01) > recurrent (02) > metastatic (06); (2) order of sample portions: higher portion numbers were selected; and (3) order of plate: higher plate numbers were selected.

#### Prioritizing functional nsSNPs in PRR pathway genes associated with IFN-I activity in cancer cells

Creating the candidate nsSNP list: We investigated five KEGG pathways within the pattern recognition receptor (PRR) network, which includes germline-encoded sensors responsible for detecting pathogen-associated molecular patterns and initiating type I interferon (IFN-I) signaling. This analysis identified 324 autosomal genes encoding proteins within the PRR pathways, which were subsequently categorized into eight functional groups. To ensure robust and reliable analysis, nsSNPs were included only if they satisfied the following criteria: (1) they were located within PRR-genes; (2) their host genes demonstrated detectable expression levels across the majority of cancer cell lines, with nsSNPs in non-expressed genes excluded; (3) their host genes exhibited significant allele frequency differences across human populations, as measured by FST values; and (4) they had sufficient genotype representation among the available cell lines to allow for statistically robust analyses. An initial set of 7,867 nsSNPs located in PRR-genes was identified. Among these, 6,446 nsSNPs corresponded to PRR-genes that were consistently expressed in more than 95% of cancer cell lines (defined as expression level FPKM >1). Of these, 2,261 nsSNPs were identified in PRR-genes exhibiting significant population-level variation (defined as a maximum F_ST_ value >0.1 within a gene). Finally, we refined the list to 71 nsSNPs in 43 PRR-genes with adequate sample representation (minor allele frequency [MAF] >0.2), facilitating robust statistical analyses.

Genotype-calling for nsSNPs in PRR genes: The genotypic profiles of each cell line (n=1,009) were determined using SNP arrays, whole-exome sequencing (WES), and computational imputation. Genotype-calling from SNP array data: Affymetrix SNP6.0 array raw data in CEL format for CCLE cell lines (n=992) were downloaded from the DepMap portal (https://depmap.org/portal/). CEL files were analyzed using Analysis Power Tools (APT) version 2.11.4 to generate normalized cross-marker signal intensity data for each cell line. The apt-probeset-genotype program from APT was applied to generate genotyping calls from the raw CEL files using the Birdseed V2 algorithm ^110^. For nsSNPs in PRR genes that were not included in the CCLE Affymetrix SNP6.0 array, imputation was performed to estimate their genotypes for each cell line (n=992). The 1000 Genomes Phase 3 project was used as the reference panel for imputation. For effective processing and computation, pre-phasing was conducted using the Segmented Haplotype Estimation and Imputation Tool (SHAPEIT) ^89^ prior to imputation. Genotype call data of CCLE cell lines from the previous steps were split into chunks, each containing a specific PRR gene. Before pre-phasing, a QC check was performed, and any problematic variants found were removed from the subsequent steps. Pre-phased data were used as input for genotype imputation by IMPUTE2 ^90^, a genotype imputation program that employs a Markov chain Monte Carlo (MCMC) algorithm, which iteratively alternates between phasing typed SNPs and imputing unobserved SNPs. Genotyping from whole exome sequencing data: For CCLE cell lines, we obtained raw sequencing data from Bioproject PRJNA523380 ^111^ through the NCBI SRA Toolkit (https://hpc.nih.gov/apps/sratoolkit.html). BAM files containing PRR genes for 325 cell lines were retrieved. Regarding TCGA specimens, raw WES data for blood-derived normal samples in all TCGA cancer types (excluding LAML) were acquired from the GDC Data Portal (https://portal.gdc.cancer.gov/). For LAML, WES data for solid tissue normal samples were used in subsequent analyses. In total, 8986 BAM files containing PRR genes across 33 TCGA cancer types were downloaded. HaplotypeCaller from GATK (Genome Analysis Toolkit, https://gatk.broadinstitute.org/hc/en-us, RRID:SCR_001876) in GVCF mode was used to generate genotype calls for nsSNPs in PRR genes. HaplotypeCaller accurately calls germline variants across multiple samples, especially for large-scale genomic projects. A Genomic Variant Call Format (GVCF) file for each sample was generated, and then joint genotyping for the whole sample cohort was performed using the GenotypeGVCFs function. Additional filtering and annotation steps, such as Variant Quality Score Recalibration (VQSR), were applied to the final VCF files. Genotyping results obtained from these three methods were then integrated, with priority given in the order of SNP array > WES > imputation.

Phenotype estimation: The intrinsic IFN-I activity of each cancer cell line was estimated by evaluating the expression of IFN-stimulated gene (ISG) signatures derived from RNA-seq profiles ^51^. ISG signatures comprise a defined set of genes that are transcriptionally activated in response to interferons. These signatures are indicative of IFN activity, as the expression levels of ISGs directly correlate with the presence and intensity of IFN signaling. Mechanistically, interferons bind to their specific cell surface receptors, initiating the JAK-STAT signaling cascade. This process leads to the activation of a transcriptional complex known as ISGF3, composed of STAT1, STAT2, and IRF9. ISGF3 subsequently binds to interferon-stimulated response elements (ISREs) within the promoters of ISGs, driving their transcription. This results in the expression of hundreds of ISG transcripts and proteins, which collectively serve as a molecular “footprint” representing the activity and extent of interferon signaling within a given cell or tissue. Genes (n = 38) used as the ISG signatures are listed in Table S15. The 38 ISG genes were retrieved from the study by Liu et al. ^51^ The ISG gene set was curated from the GSEA/mSigDB hallmark gene set collection (http://software.broadinstitute.org/gsea/msigdb/collections.jsp#H) and The Immunological Genome Project (http://www.immgen.org/).

To assess the correlation between the ISG signature and nsSNP genotypes, differentially expressed genes (DEGs) were identified between cell lines carrying reference or alternative alleles for each nsSNP using RNA-seq data from the DepMap project. Cell lines classified as fibroblasts were excluded from the analysis. DEG analysis was conducted using the R package DESeq2 with default settings, and the identified DEGs were ranked based on their log2 fold change. Gene set enrichment analysis (GSEA) was subsequently applied to evaluate whether ISG signature genes exhibited significant differences in expression between cells with distinct genotypes for each nsSNP. Pre-ranked GSEA was performed using the R package fgsea (https://github.com/ctlab/fgsea). A pre-ranked list of DEGs was used as input. The enrichment score (ES) generated by GSEA indicates the extent to which ISG signature genes are concentrated at the extremes of the ranked DEG list (either upregulated or downregulated). nsSNPs were then ranked by the absolute value of their ES, reflecting the strength of their association with the ISG signature. nsSNPs that showed no significant enrichment (adjusted p-values greater than 0.05) were eliminated from further analysis.

Multi-factor ranking: Candidate nsSNPs were prioritized based on three key factors: genetic diversity across human populations, correlations with the ISG signature, and functional evidence supporting associations with the interferon pathway. Specifically, nsSNPs exhibiting significant population diversity, stronger correlations with ISG signatures, and robust evidence for their host genes’ roles in interferon pathways were ranked higher. For population diversity, FST values were used as a quantitative metric of genetic divergence, with candidates ranked and scaled on a normalized scale from 0 to 1 using the formula: zi=xi-min(x)/max(x)-min(x), where xi is the ranked value and zi is the scaled score for the *i*th nsSNP. Correlations between genotypes and ISG signatures across cancer cell lines were analyzed using Gene Set Enrichment Analysis (GSEA), with enrichment scores (ES) similarly ranked and scaled. Functional associations with the interferon pathway were evaluated by querying PubMed using the term “gene name + interferon.” The number of publications served as a quantitative indicator of the host gene’s relevance to interferon signaling, also ranked and scaled from 0 to 1 using the same formula applied to FST values. To prioritize functionally relevant nsSNPs, we developed a composite “impact score” that integrates three rank-normalized metrics using a weighted voting strategy by assigning different weight to each of the metric. The impact score was computed as: Impact score = w_1_ × scaled ES rank + w_2_ × scaled publication rank + w_3_ × scaled FST rank, where w_1_ = 1, w_2_ = 1, and w_3_ = 0.5. Statistical significance was defined using a GSEA-based adjusted p-value < 0.05. This composite score allows for the systematic prioritization of the most functionally relevant nsSNPs for further investigation.

#### TCGA genotype data processing for genetic ancestry assessment

After merging genotype data from SNP arrays for all TCGA samples, we obtained 22,072 non-redundant samples across 33 cancer types. For patients with multiple aliquot barcodes, a single representative file was selected based on predefined criteria for both tumor and matched normal samples (see ‘SNP Array Data Collection and Processing’ section for details). Of the 909,622 SNPs genotyped on the SNP Array for TCGA, 181,946 SNPs overlapped with the processed genotype data of the reference populations (the 1000 Genomes Project and Human Genome Diversity Project [HGDP]). Quality control filtering was applied to the overlapping SNPs, requiring a call rate > 95% and a minor allele frequency (MAF) > 1%. This resulted in 150,128 high-quality bi-allelic SNPs on autosomes. These SNPs were used for downstream genetic ancestry inference. The overlapping genotype data from TCGA and the reference populations were combined and reformatted according to the input requirements of three independent ancestry inference pipelines: EIGENSTRAT, STRUCTURE, and LAMP. TCGA genotypes were strand-aligned (flipped if necessary) prior to analysis.

#### Genetic ancestry assessment by EIGENSTRAT

The genetic ancestry of TCGA patients was estimated by EIGENSTRAT ^67^, a method to study human diversity based on Principal Component Analysis (PCA) which reduces the information contained in SNP frequencies to components that capture most genetic variability, as we described before ^9^. Briefly, we used the 1000 Genomes Project as a reference and VCF files of 1000 Genome Phase 3 data were obtained from https://www.internationalgenome.org/data. Considering the reported admixture in American population in 1000 Genomes (e.g., CLM, MXL, PEL, PUR), we selected the Human Genome Diversity Project (HGDP) American population as a reference for Native American ancestry. Genotypes were formatted and quality control applied. Quality control on overlapping SNPs between TCGA and reference ensured a call rate > 95% and MAF > 1%, yielding 150,128 effective SNPs on autosomes. TCGA genotypes were flipped if needed, and combined genotype data were input into the EIGENSTRAT algorithm. The EIGENSOFT package (EIGENSTRAT algorithms included) was downloaded from GitHub (https://github.com/DReichLab/EIG). The smartpca program was applied to run PCA on the combined genotype data of TCGA and the reference populations to compute eigenvectors (by supplying a population list file using the parameter -w). The program then outputs the positions of each individual (either from TCGA or from reference populations) on the top ten axes of variation into a file with the extension.evec. This allows visualization of the population structure as well as estimation of relative distance between individuals or populations. The EIGENSOFT package, which includes EIGENSTRAT algorithms, was downloaded from GitHub (https://github.com/DReichLab/EIG). By employing the smartpca program, PCA was conducted on the merged genotype data to calculate eigenvectors (using a population list file specified by the parameter -w). The positions of each individual along the top ten axes of variation were generated by the program.

#### Genetic ancestry assessment by STRUCTURE

To estimate the global genetic ancestry for each patient in TCGA, we utilized the STRUCTURE algorithm ^93^, which infers population structure using a model-based clustering approach applied to multilocus genotype data. Version 2.3.4 of the STRUCTURE algorithm was obtained from Stanford University’s Pritchard lab website (http://web.stanford.edu/group/pritchardlab/structure.html). For our analysis, the USEPOPINFO model was employed to estimate ancestry proportions. Individuals from reference populations were assigned as representatives of continental ancestries: Northern Europeans from Utah (CEU) and Tuscans from Italy (TSI) represented European ancestry; Yoruba from Nigeria (YRI) represented African ancestry; Han Chinese (CHB) and Japanese (JPT) represented East Asian ancestry. Native American ancestry was estimated using individuals from the Human Genome Diversity Project (HGDP), specifically Colombians, Karitiana, Maya, Pima, and Surui, rather than admixed American populations from the 1000 Genomes Project, as previously described ^112^. Genome proportions originating from these ancestral populations were calculated for each individual. To enhance computational efficiency and ensure reproducibility, the analysis was repeated 10 times with 10 independent sets of 3,000 randomly selected SNPs. Final ancestry proportions were calculated as the average across all runs. In the genotype input file, ancestral reference samples were marked as “1” in the POPFLAG column, while other samples were labeled as “0.” The number of loci per run (NUMLOCI) was set to 3,000 using the -L option, and a maximum of four ancestries (European, African, East Asian, and Native American) were assumed per individual, specified using the -K option. Each STRUCTURE run included 10,000 iterations with a burn-in of 10,000 (BURNIN). Both the USEPOPINFO and PFROMPOPFLAGONLY options were set to “1” to integrate prior population information effectively.

#### STING1 local genetic ancestry assessment by LAMP

Genetic ancestries at STING1 loci for TCGA patients were estimated by the LAMP algorithm ^69^ (http://lamp.icsi.berkeley.edu/lamp/) as we described before ^9^. Specifically, we employed LAMP-ANC for the analysis. Similar to the analysis conducted with STRUCTURE, individuals from specific reference populations were used to provide prior knowledge of population-specific allele frequencies. Northern Europeans from Utah (CEU) and Tuscans from Italy (TSI) represented European ancestry, Yoruba from Nigeria (YRI) represented African ancestry, Han Chinese (CHB) and Japanese (JPT) represented East Asian ancestry, and individuals from the Human Genome Diversity Project (HGDP)—including Colombians, Karitiana, Maya, Pima, and Surui—were used to represent Native American ancestry. Given that local ancestry exhibits strong correlations among neighboring SNPs due to admixture linkage disequilibrium ^113^, chromosomal segments with consistent ancestry status were identified as ancestry blocks, with contiguous SNPs showing identical ancestry status in over 95% of patients grouped into the same block. The local ancestry of the STING1 locus for each TCGA patient was then normalized by their global ancestry estimated by STRUCTURE to reduce potential biases. Specifically, the proportion of each ancestral population at the STING1 locus for a given patient was divided by that individual’s global ancestry proportion for the corresponding ancestral population. Proportional scaling was subsequently applied to the normalized data.

#### Phylogenetic tree of the STING1 haplotypes in human

The 31 major haplotypes in human STING1 gene were inferred by Haploview (https://www.broadinstitute.org/haploview/haploview) using default setting. The haplotype-specific nucleotide sequences were aligned using multiple alignment tool Clustal Omega (https://www.ebi.ac.uk/Tools/msa/clustalo/). The resulted sequence alignments were used to construct phylogenetic trees by MEGA11^96^ (https://www.megasoftware.net/) using the minimum evolution model.

#### Assessment of tumor purity and immune infiltration in different STING1 genotypes

To evaluate the relationship between STING1 genotypes and tumor immune phenotypes, we utilized tumor purity and leukocyte fraction data from The Cancer Genome Atlas (TCGA). Tumor purity estimates were sourced from the Pan-Cancer Atlas (PanCanAtlas) project (https://gdc.cancer.gov/about-data/publications/pancanatlas), while leukocyte fraction data were retrieved from Thorsson et al. ^114^. Cancer types with fewer than five tumor samples for either the WT/WT or HAQ/HAQ genotype were excluded due to insufficient sample size. Additionally, tumors originating from hematological cells, such as acute myeloid leukemia (LAML) and diffuse large B-cell lymphoma (DLBC), were excluded as these cancers are derived from immune cells and exhibit distinct immune phenotypes. To adjust for genetic ancestry, TCGA specimens were stratified into five ancestry groups: African American (AA), European American (EA), East Asian American (EAA), Native American Ancestry (NAA), and Other Ancestry (OA). Ancestry estimation was performed using EIGENSTRAT, as described above. This stratification controlled for population-specific allele frequency differences that could confound associations between STING1 genotypes and immune phenotypes. Within each ancestry group, we performed association analyses between *STING1* genotypes (WT/WT vs. HAQ/HAQ) and tumor immune parameters (tumor purity and leukocyte fraction) using a linear regression model implemented in the limma R package (https://bioconductor.org/packages/release/bioc/html/limma.html). Cancer type was included as a covariate in each model to adjust for tumor-specific heterogeneity. Each ancestry-stratified analysis produced a p-value adjusted for cancer type. These five ancestry-specific p-values were then combined using the ‘sumlog’ function from the metap R package (https://cran.r-project.org/web/packages/metap/index.html), generating a single meta-analysis p-value that integrated information across ancestries. Immune cell infiltration data for TCGA tumors were retrieved from TIMER2.0 ^115^ (http://timer.cistrome.org). Infiltrations estimated by CIBERSORT ^68^ were used in our analysis. In each cancer type, differences in the infiltration of individual immune cells between STING1 WT and HAQ genotypes were estimated using the linear regression models in R package ‘Limma’.

#### Chemical reagents

RBN2397 (Cat No: S8993) was acquired from Selleckchem and solubilized in DMSO. 2’3’-cGAMP (Cat No: tlrl-nacga23–1) and poly (dA:dT) (Cat No: tlrl-patn-1) were procured from Invivogen and dissolved in sterile, endotoxin-free water. The DMSO concentration did not exceed 0.1% in all *in vitro* experiments.

#### Generation of STING1 isogenic cancer cell lines

LentiCRISPRv2 and lentiviral packing vectors were acquired from Addgene. Two gRNAs targeting the flanking regions of the STING1 coding sequence (CDS) were subcloned into the LentiCRISPRv2 vector. The LentiCRISPRv2 and packaging vectors were transfected into the 293T packaging cell line using the FuGENE 6 Transfection Reagent (Cat No: E2691, Promega). The medium containing lentivirus was collected 48 hours post-transfection. SK-OV-3 and TOV-21G cells were concurrently infected with lentivirus encoding two gRNAs in the presence of 8 μg/ml polybrene (Cat No: TR-1003-G, Sigma-Aldrich). Following puromycin selection (Cat No: P8833, Sigma-Aldrich), SK-OV-3 and TOV-21G cells were expanded, and single clones were isolated by limiting dilution. Genomic DNA was extracted using phenol/chloroform from each clone. PCR was conducted to verify STING1 wild type (WT) and knockout alleles, with products approximately 710 bp and 1,410 bp, respectively.

Oligos Used for gRNA Constructs:

5’ STING1 gRNA forward: 5′-CACCGAGAATCTAGACAGACGGGCG-3′

5’ STING1 gRNA reverse: 5′-AAACCGCCCGTCTGTCTAGATTCTC-3′

3’ STING1 gRNA forward: 5′-CACCGTGCTCCACACAGACACACAT-3′

3’ STING1 gRNA reverse: 5′-AAACATGTGTGTCTGTGTGGAGCAC-3′

Primers Used for PCR Verification:

STING1 knockout allele forward: 5′-TGCCATCCTAGCCTCACTCTCCA-3′

STING1 knockout allele reverse: 5′-AAGGCCCCCACTGACTCTGTCTT-3′

STING1 WT allele forward: 5′-ACCTGTGGTCTCCCTGGGTC-3′

STING1 WT allele reverse: 5′-GGCAGGGCTAGGCATCAAGG-3′

#### Generation of cell lines expressing different STING1 variants

For STING1 expression, SK-OV-3 and TOV-21G STING1 homozygous knockout clones, along with 293-Dual^™^ Null Cells lacking endogenous STING1 expression, were seeded in 12-well plates at 50% confluence one day before transfection. Transfection was conducted using 2 μg of either pMSCV-hygro-STING (RRID: Addgene_102598) or pMSCV-hygro-STING HAQ (RRID:Addgene_102600) with Lipofectamine 2000 Reagent (Cat No: 11668019, Invitrogen). The medium was changed the following day, and cells were subjected to selection with 500 μg/ml hygromycin for 4 days. Subsequently, these cells were utilized for downstream experiments.

#### DNA isolation and SNP genotyping

Genomic DNA was extracted from cancer cell lines by phenol/chloroform method. SNP genotyping was performed using rhAMP PCR. Assay probes were designed on the IDT website for STING1 SNPs, with details listed in the key resources table. Genotyping reaction reagents were purchased from IDT (Cat No: 1076016 and 1076022). rhAMP PCR was conducted in a 5 μl total volume comprising 1x rhAmp genotyping master mix, 1x rhAmp reporter mix with reference dye, and 10 ng genomic DNA. The allelic specificity of the rhAMP PCR genotyping assay was ensured by two probes, one labeled with FAM dye and the other with VIC^™^ dye (Applied Biosystems). These reporter dyes were independently detected after the PCR procedure using excitation sources and emission filters at their respective wavelengths. PCR and fluorescent readings were performed in 384-well plates on the ViiA^™^ 7 Real-Time PCR System (Applied Biosystems) following the recommended thermal cycling conditions for each genotyping platform provided by the supplier. FAM dye was collected for the reference allele, while VIC^™^ dye was collected for the alternate allele.

**Table:** 

Step	Temperature (° C)	Time (min:sec)	Cycles

Enzyme activation	95	10:00	1
Denaturation	95	00:10	40
Annealing	60	00:30	
Extension	68	00:20	
Heat inactivation	99.9	15:00	1

#### 2’3’-cGAMP stimulation

Stimulation of cells with 2’3’-cGAMP was performed using digitonin-mediated permeabilization, allowing 2’3’-cGAMP to penetrate the cell membrane and enter the cell, as previously described ^116,117^. Briefly, cells were treated with 10 μg/ml 2’3’-cGAMP in permeabilization buffer consisting of 50 mM HEPES (pH 7.4), 100 mM KCl, 3 mM MgCl2, 0.2% BSA, 85 mM sucrose, 1 mM ATP, 0.1 mM GTP, 0.1 mM DTT, and 0.001% digitonin. Following incubation at 37°C for 30 minutes, the plate wells were aspirated, and fresh complete media was added. After further incubation at 37°C for 4 hours, cells were harvested for qRT-PCR, RNA-seq, or Western blot analysis.

#### RBN2397 treatment

Cell viability analysis following RBN2397 treatment was conducted using the MTT assay (Cat No: 475989, Sigma-Aldrich) or colony formation assay. For the MTT assay, cells were seeded at a concentration of 3.0×10^4^ cells/mL in 100 μL of medium per well in 96-well plates. After overnight incubation, cells were treated with serial dilutions of RBN2397 for 72 hours. For the colony formation assay, cells were seeded at a concentration of 3.0×10^4^ cells/mL in 1 mL of medium per well in 12-well plates. After overnight incubation, cells were treated with serial dilutions of RBN2397 for 6 days. For Western blot analysis, cells treated with 0.1 μM or 1 μM RBN2397 for 24 hours were collected. Prior to treatment, cells were seeded at a concentration of 2.0×10^5^ cells/mL in 10 mL of medium per well in 10 cm-well plates. After overnight incubation, cells were treated with RBN2397 for 24 hours and then harvested for western blot analysis.

#### Quantitative Real-Time RT-PCR (qRT-PCR)

Total RNA was extracted using TRIzol Reagent (Invitrogen) and reverse-transcribed using the High Capacity RNA-to-cDNA Kit (Cat No: 4368813, Applied Biosystems) according to the manufacturer’s instructions. The cDNA was quantified by qRT-PCR using the ViiA^™^ 7 Real-Time PCR System (Applied Biosystems). PCR was performed using PowerUp SYBR Green reagents (Cat No: A25742, Applied Biosystems) as per the manufacturer’s instructions. GAPDH was utilized as an internal control. Primers used for qRT-PCR are listed below:

GAPDH

Forward: 5′-ACACCATGGGGAAGGTGAAG-3′

Reverse: 5′-AAGGGGTCATTGATGGCAAC-3′

IFNB1

Forward: 5′-TGCTCTGGCACAACAGGTAG-3′

Reverse: 5′-CAGGAGAGCAATTTGGAGGA-3′

STAT1

Forward: 5′-ATGGCAGTCTGGCGGCTGAATT-3′

Reverse: 5′-CCAAACCAGGCTGGCACAATTG-3′

CCL5

Forward: 5′-TACACCAGTGGCAAGTGCTC-3′

Reverse: 5′-CCAGACTTGCTGTCCCTCTC-3′

CXCL10

Forward: 5′-CCACGTGTTGAGATCATTGC-3′

Reverse: 5′-CCTCTGTGTGGTCCATCCTT-3′

#### RNA-sequencing

The SK-OV-3 parent cells and clones obtained from the dual CRISPR/Cas9 experiment (WT/HAQ, WT/-, and HAQ/-) were treated with 10 μg/mL of 2’3’-cGAMP for 30 minutes and then allowed to rest for 4 hours. Subsequently, cells were harvested immediately and extracted using TRIzol Reagent. Illumina mRNA TruSeq library preparation was employed, followed by sequencing on an Illumina NovaSeq platform with paired-end 150 bp reads, conducted by Azenta Life Sciences.

#### Protein isolation and western blot

Cells were lysed in mammalian protein extraction reagent (Cat No: 78505, Pierce). Following quantification using a BCA protein assay kit (Cat No: 23225, Pierce), total proteins were separated by SDS-PAGE under denaturing conditions and transferred to PVDF membranes (Cat No: IPVH00010, Millipore). The membrane was blocked in 5% blotting-grade blocker (Cat No: 1706404, Bio-Rad) and sequentially incubated with primary and secondary antibodies. Immunoreactive proteins were visualized using the Western HRP substrate (Cat No: WBLUF0500, Millipore). The antibodies used are as follows: anti-p-TBK1 (Cell Signaling Technology Cat#5483, RRID:AB_10693472), anti-TBK1 (Cell Signaling Technology Cat# 3504, RRID:AB_2255663), anti-p-STAT1 (Cell Signaling Technology Cat# 7649, RRID:AB_10950970), anti-STAT1 (Cell Signaling Technology Cat# 14994, RRID:AB_2737027), anti-β-Actin (Cell Signaling Technology Cat# 4970, RRID:AB_2223172), anti-mouse IgG HRP linked (Cell Signaling Technology Cat# 7076, RRID:AB_330924), anti-rabbit IgG HRP linked (Cell Signaling Technology Cat# 7074, RRID:AB_2099233).

#### Generation of Jurkat-CXCR3 cells

The pSBbi-RP Hu CXCR3 vector (RRID: Addgene_183255) and lentiviral packing vectors were transfected into the packaging cell line 293T using the FuGENE 6 Transfection Reagent. After 48 hours post-transfection, the medium containing lentivirus was harvested. Subsequently, Jurkat cells were infected with the lentivirus in the presence of 8 μg/ml polybrene. Following infection, puromycin selection was applied to the cells. Jurkat cells expressing dTomato were then flow cytometry sorted using a BD Biosciences Influx A, located in the Penn Cytomics & Cell Sorting Shared Resource Laboratory. Positive cells were collected and subsequently expanded, resulting in the generation of Jurkat-CXCR3 cells.

#### Transwell migration assay

Fluorescence-based transwell migration assays were employed to evaluate the migration of Jurkat-CXCR3 and NK-92 cells. Firstly, Jurkat-CXCR3 cells and NK-92 cells were labeled with 1 μM CellTracker^™^ Blue CMF2HC Dye (Cat No: C12881, Invitrogen) in medium at 37°C for 30 min. The labeled cells were then resuspended in medium containing 10% FBS to achieve a concentration of 1.0*10 ^7 cells/ml. Subsequently, the parent cells and clones obtained from the dual CRISPR/Cas9 experiment (WT/HAQ, WT/-, and HAQ/-) were treated with 5 μg/mL poly(dA:dT) for 4 hours. Following treatment, the cancer cells were resuspended in 500 μl of medium and seeded into the bottom wells of a 24-well plate. Transwell chambers with 8 μm pore filters (Cat No: 3422, Corning) were then placed into the wells. Jurkat-CXCR3 cells and NK-92 cells (1 × 10^6^ in 100 μl) were added to the chambers. After 48 hours, 72 hours, or 96 hours, the migrated cells in the bottom wells were collected. Immune cells exhibiting fluorescence were quantified using a BD Biosciences Influx A.

#### Enzyme-linked immunosorbent assay (ELISA) for IFN-β

The STING1 homozygous knockout clones of SK-OV-3 and TOV-21G cells were obtained from a dual CRISPR/Cas9 experiment. To induce STING1 overexpression, cells were seeded in 12-well plates at a confluence of 50% one day before transfection. Transfection was conducted with 2 μg of pMSCV-hygro-STING (Cat No: 102598, Addgene) or 2 μg of pMSCV-hygro-STING HAQ (Cat No: 102600, Addgene) using Lipofectamine 2000 Reagent (Cat No: 11668019, Invitrogen). The medium was changed the next day, and cells were selected with 500 μg/ml hygromycin for 4 days. To measure Human IFN-β production, cells expressing different subtypes of STING1 (WT and HAQ) were treated with 10 μg/mL of 2’3’-cGAMP for 30 minutes followed by a 4-hour rest period. Cell culture supernatants were collected, and Human IFN-beta DuoSet ELISA kit (Cat No: DY814–05, R&D Systems) was utilized to measure IFN-β production as per the manufacturer’s instructions. Additionally, the cells were directly lysed, and western blot analysis was performed to verify the expression of STING1 and β-Actin.

#### Interferon reporter assay

293-Dual^™^ Null Cells lack endogenous STING1 expression. For STING1 overexpression, these cells were seeded in 12-well plates at a confluence of 50% one day before transfection. Transfection was carried out using 2 μg of pMSCV-hygro-STING (Cat No: 102598, Addgene) or 2 μg of pMSCV-hygro-STING HAQ (Cat No: 102600, Addgene) with Lipofectamine 2000 Reagent (Cat No: 11668019, Invitrogen). The medium was changed the next day, and cells were selected with 500 μg/ml hygromycin for 4 days before seeding for the interferon reporter assay. Subsequently, 293-Dual^™^ Null Cells expressing different subtypes of STING1 (WT and HAQ) were treated with 10 μg/mL of 2’3’-cGAMP for 30 minutes followed by a 4-hour rest period. Cell culture supernatants were collected to measure luciferase activity using the QUANTI-Luc^™^ 4 Lucia/Gaussia kit (Invivogen) and SEAP level using the QUANTI-Blue^™^ Solution (Invivogen). In addition, the cells were directly lysed, and western blot analysis was performed to verify the expression of STING1 and β-Actin.

### QUANTIFICATION AND STATISTICAL ANALYSIS

Statistical analyses were performed using RStudio. Categorical variables were analyzed using Fisher’s exact test. Differences between two groups of continuous variables were assessed using Student’s t-test or the Wilcoxon rank-sum test, depending on data distribution. Results are reported as mean ± SD. Significance was noted in figures or indicated by * p value < 0.05, ** p value < 0.01, as noted in the corresponding figure legends.

## Supplementary Material

1

2

3

4

5

6

7

8

10

11

12

13

14

15

16

17

SUPPLEMENTAL INFORMATION

Supplemental information can be found online at https://doi.org/10.1016/j.celrep.2025.116882.

## Figures and Tables

**KEY RESOURCES TABLE:** 

REAGENT or RESOURCE	SOURCE	IDENTIFIER
Antibodies		
Phospho-TBK1/NAK (Ser172)	Cell Signaling Technology	Cat #5483; RRID: AB_2737027
TBK1/NAK (D1B4)	Cell Signaling Technology	Cat #3504; RRID: AB_2255663
Phospho-Stat1 (Tyr701)	Cell Signaling Technology	Cat #7649; RRID: AB_10950970
Stat1 (D1K9Y)	Cell Signaling Technology	Cat #14994; RRID: AB_2737027
STING (D2P2F)	Cell Signaling Technology	Cat #13647; RRID: AB_2732796
β-Actin	Cell Signaling Technology	Cat #4970; RRID: AB_2223172
Anti-mouse IgG	Cell Signaling Technology	Cat #7076; RRID: AB_330924
Anti-rabbit IgG	Cell Signaling Technology	Cat #7074; RRID: AB_2099233
Chemicals, peptides, and recombinant proteins		
RBN2397	Selleckchem	Cat# S8993
2’3’-cGAMP	Invivogen	Cat# tlrl-nacga23-1
poly(dA:dT)	Invivogen	Cat# tlrl-patn-1
FuGENE 6	Promega	Cat# E2691
Lipofectamine 2000	Invitrogen	Cat#11668019
MTT	Sigma-Aldrich	475989
Crystal violet solution	Sigma-Aldrich	HT901
Polybrene infection/transfection reagent	Sigma-Aldrich	TR-1003-G
Puromycin	Sigma-Aldrich	P8833
Hygromycin	Roche	10843555001
Blotting-grade blocker	Bio-Rad	1706404
Immobilon forte western HRP substrate	Millipore Sigma	WBLUF0500
CellTracker^™^ Blue CMF2HC Dye	Invitrogen	C12881
Critical commercial assays		
rhAmp^®^ Genotyping Master Mix	IDT	Cat#1076016
rhAmp^®^ Reporter Mix w/Reference	IDT	Cat#1076022
STING1 R71H rs11554776	IDT	Cat# CD.GT.KJQS7656.1
STING1 G230A rs78233829	IDT	Cat# Hs.GT.rs78233829.G.1
STING1 R232H rs1131769	IDT	Cat# CD.GT.HPNL6434.1
STING1 R293Q rs7380824	IDT	Cat# Hs.GT.rs7380824.T.1
High-Capacity RNA-to-cDNA Kit	Applied Biosystems	Cat#4368813
PowerUp SYBR Green	Applied Biosystems	Cat# A25742
Mammalian protein extraction reagent	Pierce	Cat# 78505
BCA protein assay kit	Pierce	Cat# 23225
Transwell Permeable Supports	Corning	Cat# 3422
Human IFN-beta DuoSet ELISA kit	R&D Systems	Cat# DY814-05
QUANTI-Blue^™^ Solution	Invivogen	Cat# rep-qbs
QUANTI-Luc^™^ 4 Lucia/Gaussia	Invivogen	Cat# rep-qlc4lg1
Deposited data		
1000 Genomes Phase 3 data	1000 Genomes project	https://www.internationalgenome.org/data-portal/data-collection/grch38
SNP array data for TCGA	TCGA project	https://gdc-portal.nci.nih.gov
RNAseq data for TCGA	UCSC Toil RNAseq Recompute Compendium	https://xenabrowser.net/datapages/?hub=https://toil.xenahubs.net:443
Segmentation data for TCGA	the GDAC Firehose of the Broad Institute	http://gdac.broadinstitute.org/
Mutation profiles for TCGA	TCGA Multi-Center Mutation Calling in Multiple Cancers (MC3) project	https://doi.org/10.7303/syn7214402
Fusion profiles for TCGA	TumorFusions data portal	http://tumorfusions.org/
Genomic profiles for cancer cell lines	the Dependency Map (DepMap) portal	https://depmap.org/portal/
CRISPR for cancer cell lines	the Score projects	https://doi.org/10.6084/m9.figshare.c.5289226.v1
TCGA tumors infiltration	TIMER2.0	http://timer.cistrome.org
RNA sequencing	This paper	GEO: GSE314074
Data generated in this study	This paper	http://52.25.87.215/IDmap/
Experimental models: Cell lines		
293T	ATCC	Cat #CRL-3216; RRID: CVCL_0063
CAL27	ATCC	Cat #CRL-2095; RRID: CVCL_1107
NK-92	ATCC	Cat #CRL-2407; RRID: CVCL_2142
Jurkat	ATCC	Cat #TIB-152; RRID: CVCL_0065
HCC1143	ATCC	Cat #CRL-2321; RRID: CVCL_1245
NCI-H1373	ATCC	Cat #CRL-5866; RRID: CVCL_1465
BHY	DSMZ	Cat #ACC-404; RRID: CVCL_1086
293-Dual^™^ Null Cells	InvivoGen	Cat #293d-null
TOV-21G	ATCC	Cat # CRL-3577; RRID: CVCL_3613
SK-OV-3	NCI Development Therapeutics Program	RRID: CVCL_0532
HARA	JCRB	Cat # JCRB1080.0; RRID: CVCL_2914
SNG-M	JCRB	Cat # JCRB0179; RRID: CVCL_1707
A-253	ATCC	Cat# HTB-41
Caov-3	ATCC	Cat# HTB-75
Detroit 562	ATCC	Cat# CCL-138
DU 145	ATCC	Cat# HTB-81
FaDu	ATCC	Cat# HTB-43
HCC1937	ATCC	Cat# CRL-2336
HCC1954	ATCC	Cat# CRL-2338
HCC38	ATCC	Cat# CRL-2314
Hs 578T	ATCC	Cat# HTB-126
HT-29	ATCC	Cat# HTB-38
LoVo	ATCC	Cat# CCL-229
MDA-MB-231	ATCC	Cat# HTB-26
MDA-MB-361	ATCC	Cat# HTB-27
MES-SA	ATCC	Cat# CRL-1976
OVCAR-8	NCI Development Therapeutics Program	RRID:CVCL_1629
PEO1	Sigma Aldrich	Cat# 10032308
SCC-9	ATCC	Cat# CRL-1629
SCC-15	ATCC	Cat# CRL-1623
SCC-25	ATCC	Cat# CRL-1628
SK-BR-3	ATCC	Cat# HTB-30
SK-CO-1	ATCC	Cat# HTB-39
SW 620	ATCC	Cat# CCL-227
U-87 MG	ATCC	Cat# HTB-14
UPCI-SCC-040	DSMZ	Cat# ACC-660
UPCI-SCC-074	DSMZ	Cat# ACC-664
UWB1.289	ATCC	Cat# CRL-2945
Oligonucleotides		
5’ STING1 gRNA forward: 5′-CACCGAGAATCTAGACAGACGGGCG-3′	This paper	NA
5’ STING1 gRNA reverse: 5′-AAACCGCCCGTCTGTCTAGATTCTC-3′	This paper	NA
3’ STING1 gRNA forward: 5′-CACCGTGCTCCACACAGACACACAT-3′	This paper	NA
3’ STING1 gRNA reverse: 5′-AAACATGTGTGTCTGTGTGGAGCAC-3′	This paper	NA
STING1 knockout allele forward: 5′-TGCCATCCTAGCCTCACTCTCCA-3′	This paper	NA
STING1 knockout allele reverse: 5′-AAGGCCCCCACTGACTCTGTCTT-3′	This paper	NA
STING1 WT allele forward: 5′-ACCTGTGGTCTCCCTGGGTC-3′	This paper	NA
STING1 WT allele reverse: 5′-GGCAGGGCTAGGCATCAAGG-3′	This paper	NA
Forward primer for CXCL10: 5′-CCACGTGTTGAGATCATTGC-3′	This paper	NA
Reverse primer for CXCL10: 5′-CCTCTGTGTGGTCCATCCTT-3′	This paper	NA
Forward primer for CCL5: 5′-TACACCAGTGGCAAGTGCTC-3′	This paper	NA
Reverse primer for CCL5: 5′-TACACCAGTGGCAAGTGCTC-3′	This paper	NA
Forward primer for STAT1: 5′-ATGGCAGTCTGGCGGCTGAATT-3′	This paper	NA
Reverse primer for STAT1: 5′-CCAAACCAGGCTGGCACAATTG-3′	This paper	NA
Forward primer for IFNB1: 5′-TGCTCTGGCACAACAGGTAG-3′	This paper	NA
Reverse primer for IFNB1: 5′-CAGGAGAGCAATTTGGAGGA-3′	This paper	NA
Forward primer for GAPDH: 5′-ACACCATGGGGAAGGTGAAG-3′	This paper	NA
Reverse primer for GAPDH: 5′-AAGGGGTCATTGATGGCAAC-3′	This paper	NA
Recombinant DNA		
LentiCRISPRv2	Sanjana et al.^83^	Addgene #52961
pMSCV-hygro-STING	Cerboni et al.^84^	Addgene #102598
pMSCV-hygro-STING HAQ	Cerboni et al.^84^	Addgene #102600
pSBbi-RP Hu CXCR3	Unpublished	Addgene #183255
Software and algorithms		
SnpEff	Cingolani et al.^85^	https://pcingola.github.io/SnpEff/
popgenome	Pfeifer et al.^86^	https://github.com/pievos101/PopGenome
Picard	‘‘Picard Toolkit.’’ 2019. Broad Institute, GitHub Repository	https://broadinstitute.github.io/picard/
GISTIC	Mermel et al.^87^	https://www.broadinstitute.org/cancer/cga/gistic
STAR	Dobin et al.^88^	https://github.com/alexdobin/STAR
Analysis Power Tools (APT) version 2.11.4		https://www.thermofisher.com/us/en/home/life-science/microarray-analysis/microarray-analysis-partners-programs/affymetrix-developers-network/affymetrix-power-tools.html
Segmented Haplotype Estimation and Imputation Tool (SHAPEIT)	Delaneau et al.^89^	https://mathgen.stats.ox.ac.uk/genetics_software/shapeit/shapeit.html
IMPUTE2	Howie et al.^90^	NA
SRA Toolkit	NCBI	https://hpc.nih.gov/apps/sratoolkit.html
GATK	Poplin et al^91^	https://gatk.broadinstitute.org/hc/en-us, RRID:SCR_001876
R package ‘fgsea’	Korotkevich et al.^92^	https://github.com/ctlab/fgsea
EIGENSTRAT	Price et al.^67^	https://github.com/DReichLab/EIG
STRUCTURE	Pritchard et al.^93^	https://web.stanford.edu/group/pritchardlab/structure.html
LAMP	Sankararaman et al.^69^	http://lamp.icsi.berkeley.edu/lamp/
Haploview	Barrett et al.^94^	https://www.broadinstitute.org/haploview/haploview
Clustal Omega	Madeira et al.^95^	https://www.ebi.ac.uk/Tools/msa/clustalo/
MEGA11	Tamura et al.^96^	https://www.megasoftware.net/
DESeq2	Love et al.^97^	https://bioconductor.org/packages/release/bioc/html/DESeq2.html

## Highlights

- Immune genes show increased nsSNP variation, reflecting the evolution of the immune system
- Cancer genes exhibit lower nsSNP variation, indicating strong evolutionary conservation
- nsSNPs in genes linked to PRR pathways are associated with diverse intrinsic IFN-I activity
- *STING1* gene nsSNPs vary widely among populations and are associated with IFN-I activity
