# Supplementary material for: Immune gene diversity and STING1 variants in shaping cancer immunity across different genetic ancestry populations
Source: Cell Rep. Author manuscript; Available in PMC 2026 Mar 26. (PMC13019158; doi:10.1016/j.celrep.2025.116882)
Supplement: 1 [file NIHMS2151699-supplement-1.pdf]

Cell Reports, Volume *45*

Supplemental information

Immune gene diversity and *STING1* variants in shaping cancer immunity across different genetic ancestry populations

Xiaowen Hu, Jie Huang, Jiao Yuan, Yanrong Sun, Yuxin Wang, Zhongyi Hu, Junjie Jiang, Zhiling Wang, Bingwei Wang, Meixiao Long, Kara N. Maxwell, Yi Fan, Janos L. Tanyi, Kathleen T. Montone, Hongzhe Li, Sarah H. Kim, Katherine L. Nathanson, Timothy R. Rebbeck, Susan M. Domchek, Robert H. Vonderheide, and Lin Zhang

Supplementary Figures

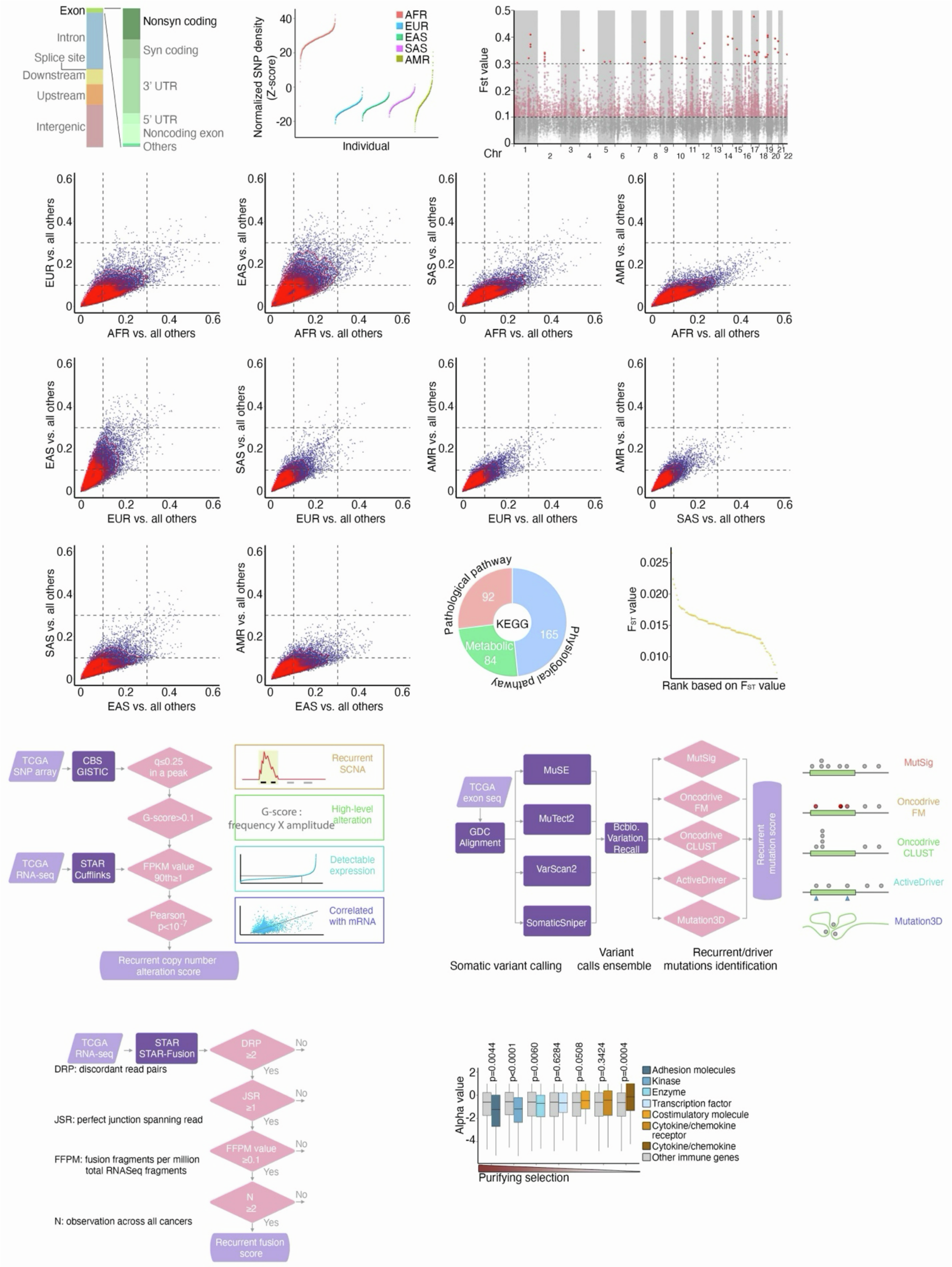

Figure S1. Immune-related genes exhibit increased FST values for nsSNPs, whereas cancer-related genes display lower FST values

A. SNP fractions are distributed across various components of the human genome (left), with exonic SNPs further categorized into six subgroups (right). **B.** Normalized nsSNPs density scores (Z-scores) per genome. Each dot corresponds to an individual and reflects the nsSNP density that normalized by gene size and chromosomal location. Each dot is colored by its corresponding ancestry. **C.** The distribution of global F_{ST} values for genes containing nsSNPs across the human genome is represented by colored dots: red >0.3; pink between 0.1 and 0.3; gray <0.1. **D.** A density plot shows the comparation of F_{ST} values between each super population against all other super populations. **E.** Overview of the KEGG pathways (n=341) analyzed in this study. **F.** The bubble plots show mean F_{ST} values for each pathway are depicted across metabolic pathways, from left to right. Pathways within each group are ranked by the mean F_{ST} value. Bubble size represents the number of genes in each pathway, and color denotes selected pathways. Upper panels display gene set enrichment analysis results for pathways significantly enriched for higher or lower F_{ST} values. **G.** Computational approach for estimating recurrent scores for somatic copy number alteration. The detailed computational approach was described in our previous publications as well as in the Methods section. **H.** Computational approach for estimating recurrent scores for somatic mutation. **I.** Computational approach for estimating recurrent scores for transcript fusion. **J.** Box plot shows the alpha values of KEGG immune pathway genes across different functional groups.

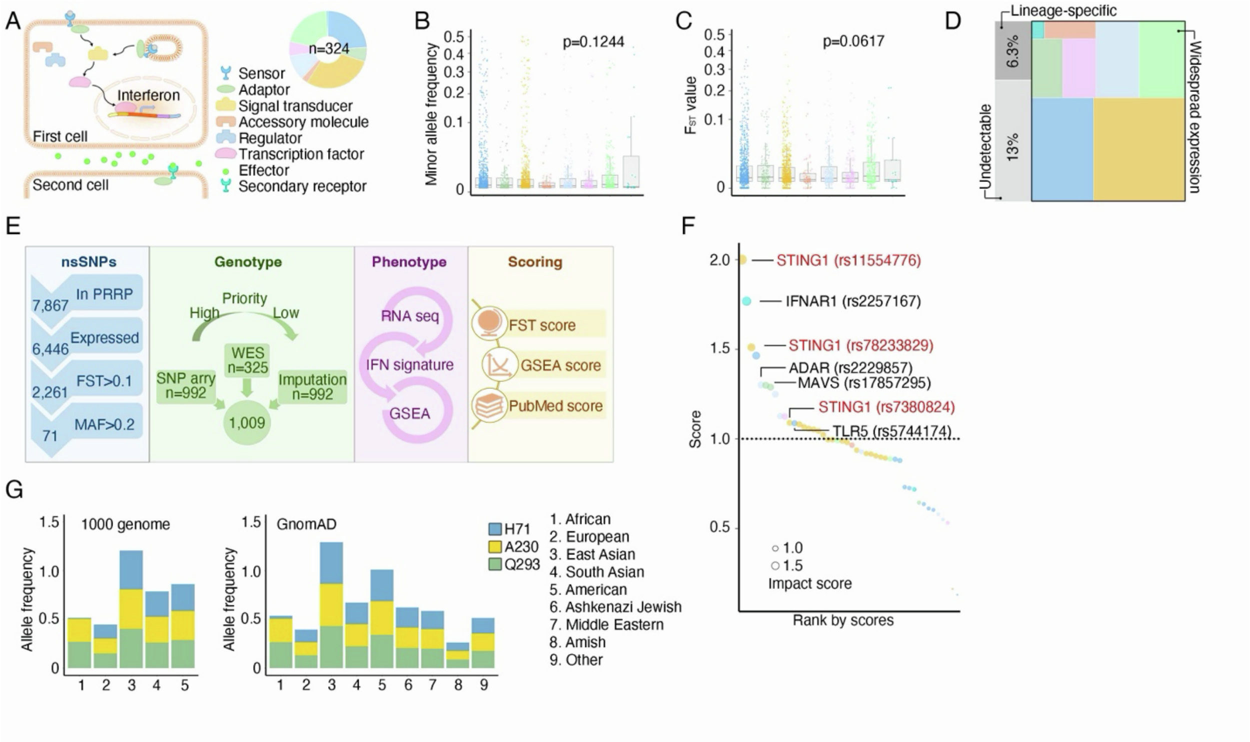

Figure S2. nsSNPs in PRR pathway genes are associated with diverse intrinsic interferon activity in cancer cells

A. Illustration of the PRR pathways along with functional classification of the genes in these pathways. **B.** Box plot represents the minor allele frequencies of the PRR-Gene genes across the eight functional categories. **C.** A box plot represents the F_{ST} value of the PRR-Gene genes across the eight functional categories. **D.** A mosaic plot illustrates the distribution of the PRR-Gene genes based on their expression patterns. **E.** Illustration of a four-step workflow to identify nsSNPs within PRR-Gene genes that potentially affect intrinsic IFN-I signaling in cancer cell lines. **F.** Impact scores for the top-ranked nsSNPs (impact score >1) and their corresponding genes after adjusting for tissue types. Each nsSNP is ranked based on its impact score. The size of the bubble denotes the level of the impact score, and the color signifies their functional category. **G.** Frequency of identified nsSNPs in *STING1* gene across the 1000 genome (left) and gnomAD (right) cohorts.

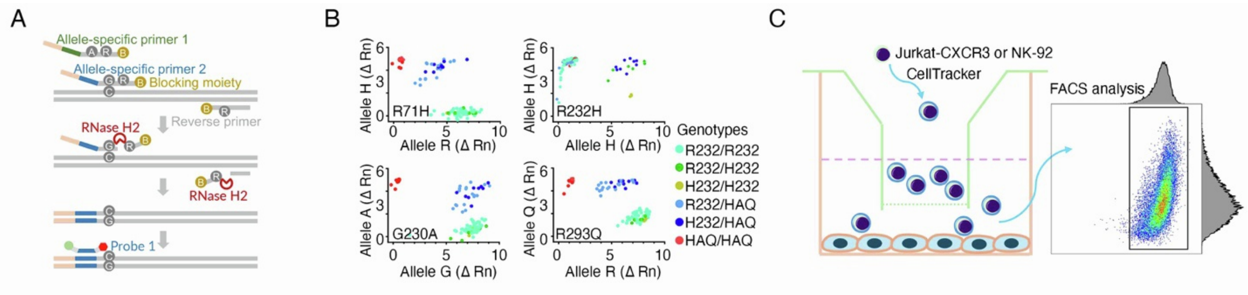

Figure S3. *STING1* variants functionally influenced the intrinsic cGAS-STING1-IFN signaling in cancer cells

A. Illustration of rhPCR genotyping technology. Two allele-specific primers and a common reverse primer are designed to target the SNP region and specifically bind to complementary DNA strands. The RNase H2 enzyme cleaves primers that have perfectly matched target sequences, removing the RNA base and 3′ blocking modification, enabling extension by Taq Polymerase. In the first two cycles, a tail sequence is added, detected by a universal probe-based system. Polymerase extension degrades the probe, generating a signal. **B.** The genotyping results of four *STING1* nsSNPs in a panel of cancer cell lines (n=34) using rhPCR-based allelic discrimination. The normalized reporter signal (Rn) for allele 1 (FAM) and allele 2 (VIC) were plotted on the X- and Y-axes, respectively. The allelic discrimination plot shows three distinct genotype clusters, including individuals homozygous for the reference allele (depicted in red), heterozygous (shown in green), and those homozygous for the alternate allele (represented in blue). **C.** Illustration of the transwell migration assay of T cells (Jurkat-CXCR3) and NK cells (NK92) to cancer cells.

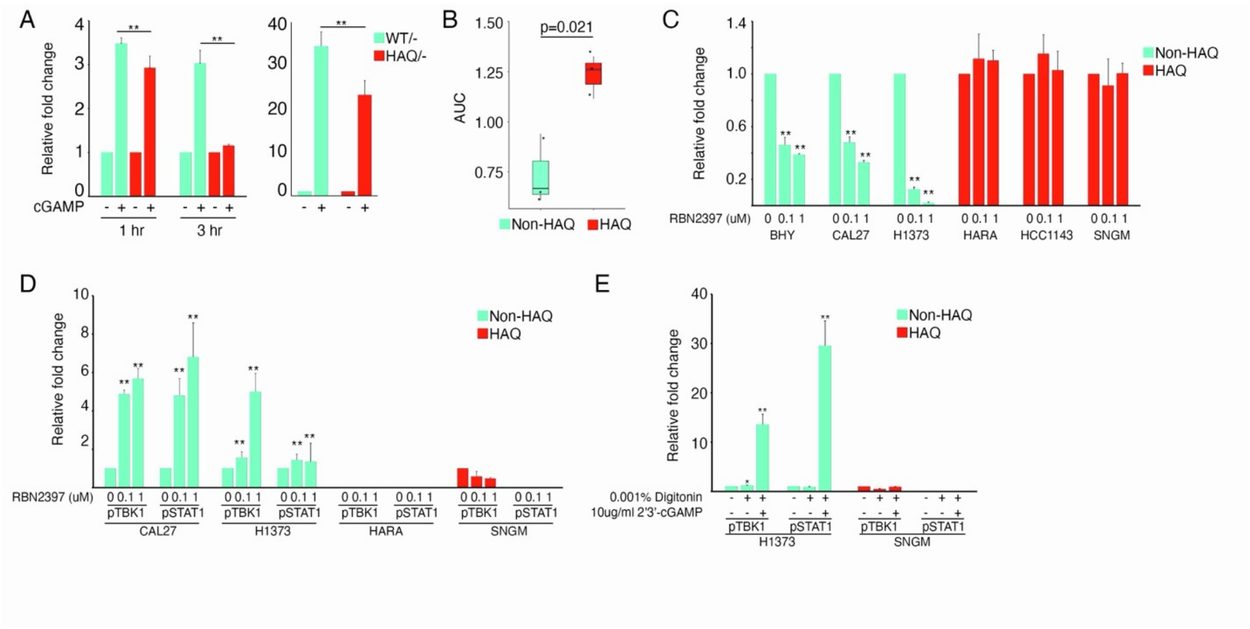

Figure S4. Quantification results for statistical analysis.

A. Quantification of western blot results for pTBK1 (left) and pSTAT1 (right) in Figure 3E. **B.** Quantification results for MTT assay in Figure 4I. **C.** Quantification results for colony formation assay in Figure 4J. **D.** Quantification results for western blot in Figure 4K. **E.** Quantification results for western blot in Figure 4L. Data are represented as mean ± SD. * represents p value <0.05, and ** represents p value <0.01.

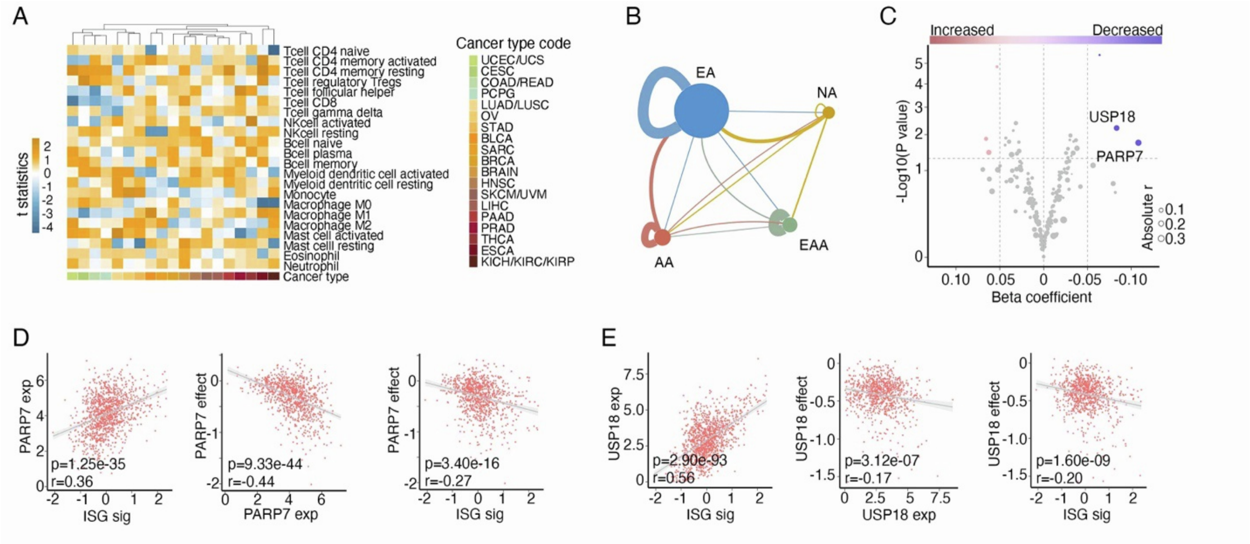

Figure S5. *STING1* variants are associated with diverse tumor immunity and treatment responses targeting the intrinsic IFN-I signal

A. Heatmap shows differences in immune cell infiltrations between TCGA specimens with *STING1* WT/WT and HAQ/HAQ haplotypes. **B.** The relationship between the global genetic ancestry of each TCGA patient and their local ancestry at the *STING1* location. The size of the circle represents the sample number in each population. Line connections represent the relation between global ancestry and local ancestry at the *STING1* genomic location. Line width indicates the percentage of samples in this population whose *STING1* local ancestry belongs to the population it connected to. **C.** Volcano plot illustrates the differences in gene dependencies within the IFN-I pathway between *STING1* WT and HAQ cancer cells. Genes showing trends of decreased and increased dependencies in HAQ cells are represented in blue and pink, respectively. The size of the genes indicates the correlations of their expression with the ISG signature. **D.** Correlations between *PARP7* mRNA expression and the ISG signature (left), the effect of PARP7 CRISPR screen and its expression (middle), and the ISG signature (right) in Depmap cancer cell lines. Note: a strong negative effect (e.g., reduced viability) upon gene knockout indicates a high dependency on the gene. **E.** Correlations between *USP18* mRNA expression and the ISG signature (left), the effect of USP18 CRISPR screen and its expression (middle), and the ISG signature (right) in Depmap cancer cell lines. Note: a strong negative effect (e.g., reduced viability) upon gene knockout indicates a high dependency on the gene.
